# Merger mania: mergers and acquisitions in the generic drug sector from 1995 to 2016

**DOI:** 10.1186/s12992-017-0285-x

**Published:** 2017-08-22

**Authors:** Marc-André Gagnon, Karena D. Volesky

**Affiliations:** 1 Associate Professor, School of Public Policy and Administration, Carleton University, Richcraft Hall, Room 5201, 1125 Colonel By Drive, Ottawa, ON K1S 5B6 Canada; 2 Department of Epidemiology, Biostatistics and Occupational Health, McGill University, 1020 Pine Avenue West, Montreal, QC H3A 1A2 Canada

**Keywords:** Pharmaceuticals, Generics, Drugs, Mergers, Acquisitions, Shortages, Pricing

## Abstract

**Background:**

Drug shortages and increasing generic drug prices are associated with low levels of competition. Mergers and acquisitions impact the level of competition. Record merger and acquisition activity was reported for the pharmaceutical sector in 2014/15, yet information on mergers and acquisitions in the generic drug sector are absent from the literature. This information is necessary to understand if and how such mergers and acquisitions can be a factor in drug shortages and increasing prices.

**Methods:**

Data on completed merger and acquisition deals that had a generic drug company being taken over (i.e. ‘target’) were extracted from Bloomberg Finance L.P. The number and announced value of deals are presented globally, for the United States, and globally excluding the United States annually from 1995 to 2016 in United States dollars.

**Results:**

Generic drug companies comprised 9.3% of the value of all deals with pharmaceutical targets occurring from 1995 to 2016. Globally, in 1995 there were no deals, in 2014 there were 22 deals worth $1.86 billion, in 2015 there were 34 deals totalling $33.56 billion, and in 2016 there were 42 deals worth in excess of $44 billion. This substantial increase was partially attributed to Teva’s 2016 acquisition of Allergan’s generic drug business. The surge in mergers and acquisitions for 2015/16 was driven by deals in the United States, where they represented 89.7% of the dollar value of deals in those years.

**Conclusions:**

The recent blitz in mergers and acquisitions signals that the generic drug industry is undergoing a transformation, especially in the United States. This restructuring can negatively affect the level of competition that might impact prices and shortages for some products, emphasizing the importance of updating regulations and procurement policies.

**Electronic supplementary material:**

The online version of this article (doi:10.1186/s12992-017-0285-x) contains supplementary material, which is available to authorized users.

## Background

In the wake of expired patents and the introduction of biosimilars, the $61 billion generic drug manufacturing industry in the United States is expected to continue growing [1]. Generic drugs (including biosimilars) accounted for 89% in the United States of all dispensed prescription drugs in 2016, up from 72% in 2008 and 43% in 1996 [2]. Since generic drugs are priced lower than their branded counterparts, they generate cost savings for individuals and drug plans [1, 3].

Record numbers of mergers and acquisitions in the pharmaceutical industry were reported for the years 2014 and 2015, based on the announcement date of the deals [4, 5]. The literature has cited several potential reasons for pharmaceutical companies pursuing mergers and acquisitions. They include: achieving economies of scale and scope, gaining corporate control, acquiring specific assets such as patents, and buying out dying or financially weak companies [1, 6]. The literature on mergers and acquisitions has typically focused on the brand-name pharmaceutical sector and the relationship between mergers and acquisitions and research and development or productivity [7–10], which is not relevant to understanding the dynamics in the generic drug sector. In the case of generics, we find little literature on the causes, impacts, and magnitude of mergers and acquisitions in that sector.

Mergers and acquisitions in the generic sector are often considered a business decision to increase efficiency gains [4]. However, studies analyzing increasing prices of generics and drug shortages have observed that mergers and acquisitions were often a factor associated with significant price increases, drug shortages, supply disruption, and a reduced number of competing manufacturers [3, 11–17]. Increasing generic drug prices and drug shortages have become pressing issues particularly in the United States [2, 13]. Before 2013, price increases for generic drugs were less significant in the United States, while since 2013 changes in these drugs’ prices substantially increased overall drug spending [14]. According to a 2014 study by the Drugs Channel Institute and Pembroke Consulting, the price of half of the generic drugs available in the United States increased from the previous 12 months [3]. A study of 1120 generic drugs demonstrated that drugs with fewer suppliers were more likely to be associated with price increases. Generics with a duopoly, near-monopoly, and monopoly were associated with price increases of 29%, 59% and 116% respectively between 2008 and 2013 as compared to drugs with the highest level of competition [17]. While increases in generic drug prices and shortages are related to market competition levels, mergers and acquisitions carry the risk of decreasing competition [16, 17].

The few studies on merger and acquisition activity in the pharmaceutical drug sector over time provide little information on the most recent trends in terms of the volume or geographic breakdown of this activity and provide no clear presentation of the methods used to analyze the trends [18, 19]. Additionally, because the impact of mergers and acquisitions can be observed after their completion and not at the time of the announcement, it is important to compile mergers and acquisitions based on the completion date, which has not been done previously for the generic drug sector. As reports indicate that 2014 and 2015 were landmark years in terms of mergers and acquisitions involving pharmaceutical companies (based on the date of the deals’ announcement), further investigation into the extent of merger and acquisition activity in the generic sector will provide important information on its present state and indications of its future directions. This study measures the magnitude of mergers and acquisitions in the generic pharmaceutical sector in the United States and abroad from 1995 to 2016.

## Methods

To study merger and acquisition deals in the generic drug sector, we examined the number and size of completed deals over time that had a target firm (i.e. firm being taken over) classified as a generic medical drug company. We focused on completed deals, rather than pending or proposed ones, because the potential impacts of the deal start to come to fruition after completion. To identify the largest mergers and acquisitions that might have impacted the data for specific years, the ten largest deals in terms of the announced dollar value were reported.

### Data

**Fig. 1:**
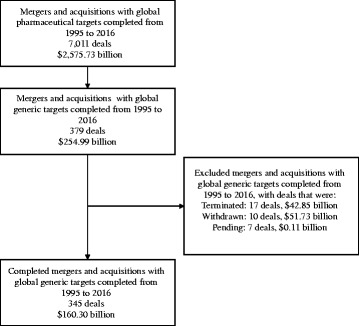
Flowchart of how study sample was selected. Dollar values are in United States dollars and represent the total announced value of merger and acquisition deals

Bloomberg Finance L.P. was used to identify and collect information on mergers and acquisitions in the pharmaceutical sector. Bloomberg L.P. terminal is a financial software that assembles real-time data on markets. To obtain information on mergers and acquisitions in the generic medical drug sector, ‘MA’ was searched and the data were filtered by select attributes. Deals included in the analysis were: classified as a merger and/or acquisition, and had a target firm classified as a medical drug – generic sector firm, and were considered completed deals. Results for generic targets based in any region (referred to as global) were compiled according to the deals’ completion date for each year from January 1st, 1995 to December 31st, 2016. This process was repeated with generic targets based in the United States and then with global deals excluding the United States. Figure 1 demonstrates how the data were selected.

Not all firms publically release the announced value paid to acquire another firm. Specifically, when private companies are acquired, the value and other financial terms of the deal do not have to be disclosed. Larger companies are often public companies and are required to disclose the financial terms of the merger and acquisition deal. As a result, some deals do not have an announced dollar value available and are only represented in the number of deals. All figures are presented in United States dollars and were not adjusted for inflation.

### Analysis

To summarize the data, line graphs display the number of deals and total announced value for each year from 1995 to 2016. The number of deals reflects both those with released and private announced values. The top ten deals in terms of highest announced value over the 21 years under study are tabulated.

## Results

The final study sample consisted of 345 deals totalling $160.30 billion in value (Fig. 1). Globally, merger and acquisition deals from 1995 to 2016 with generic drug targets represented 9.3% of the total value of mergers and acquisitions with pharmaceutical targets (Table 1). From 1995 to 2016 combined, generic targets in the United States made up the majority (63.5%) of the value of deals with generic targets.

**Table 1:** Total announced value of merger and acquisition deals with pharmaceutical and generic drug targets ^a,b,c^

Sector		Global	United States	Global excluding the United States
All pharmaceuticals from 1995 to 2016 (in billions)		$1717.69	$1035.57	$682.12
Generics from 1995 to 2016		$160.30	$101.71	$58.59
Generics as % of total		9.3%	9.8%	8.6%
All pharmaceuticals	2014	$105.14	$64.92	$40.22
	2015	$250.32	$191.20	$59.12
	2016	$126.28	$97.43	$28.85
Generics	2014	$1.86	$0.89	$0.97
	2015	$33.56	$26.66	$6.90
	2016	$44.01	$42.95	$1.06
Generics as % of all pharmaceuticals	2014	1.8%	1.4%	2.4%
	2015	13.4%	14.0%	11.6%
	2016	34.9%	44.1%	3.7%

^a^Data were gathered from Bloomberg L.P. Finance

^b^Included completed deals with a generic target

^c^Figures are in billions of United States dollars and were not adjusted for inflation

**Fig. 2:**
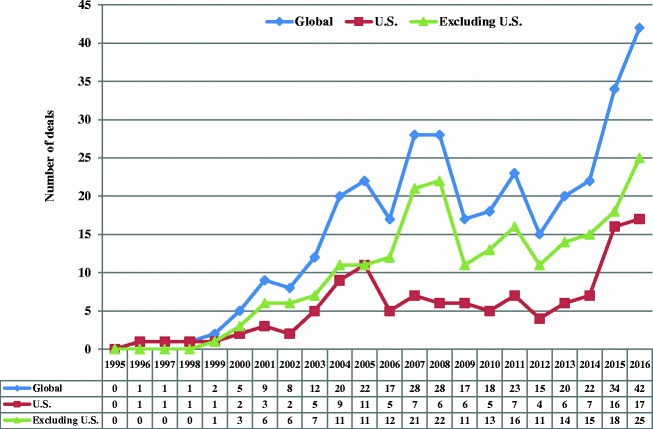
Number of completed merger and acquisition deals with generic targets from 1995 to 2016. Data were gathered from Bloomberg Finance L.P. The number of deals included deals that did not release the announced value

**Fig. 3:**
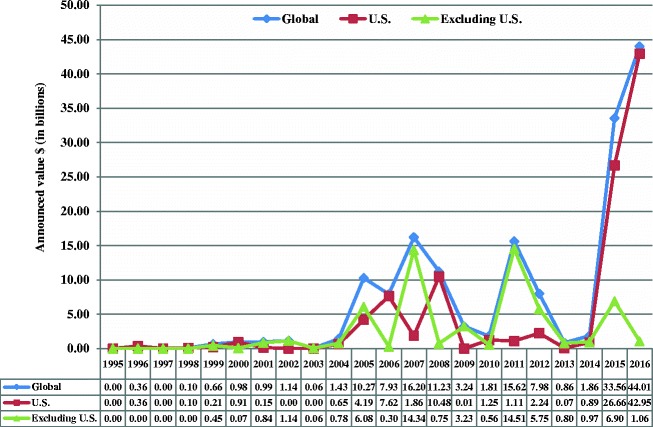
Total announced value of completed deals with generic targets from 1995 to 2016. Data were gathered from Bloomberg Finance L.P. terminal. Financial figures are in billions of United States dollars not adjusted for inflation and rounded to two decimal places

In 1995, the total value of mergers and acquisitions with generic targets was negligible, and in 2016 the annual value was $44.01 billion, representing 34.9% of all mergers and acquisitions in the pharmaceutical sector. While the number of deals had substantially increased since 2000, the total announced value of the deals increased even more (Figs. 2 and 3). There were 158, 54, and 104 completed deals without available announced values for global, United States and global excluding United States regions respectively (data not shown).

Table 2 provides a list of the 10 largest merger and acquisition deals with generic targets by dollar amount. The increase in value from 2014 to 2016 is partially explained by a higher volume of deals, but one deal in particular, Teva’s acquisition of Allergan’s generic drug business, represented 89.9% of the total value for 2016.

**Table 2:** 10 largest global deals with generic targets from 1995 to 2016 ^a,b,c^

Target	Acquirer	Announced value (in billions)	Announced date	Completion date
Allergan’s generic drug business	Teva Pharmaceutical Industries Ltd	39.56	27 July 2015	2 August 2016
Hospira Inc	Pfizer Inc	16.81	5 February 2015	3 September 2015
Nycomed A/S	Takeda Pharmaceutical Co Ltd	13.73	19 May 2011	30 September 2011
Barr Pharmaceuticals Inc	Teva Pharmaceutical Industries Ltd	8.83	18 July 2008	23 December 2008
Par Pharmaceutical Holdings Inc	Endo International PLC	8.09	18 May 2015	28 September 2015
IVAX Corp	Teva Pharmaceutical Industries Ltd	7.58	25 July 2005	26 January 2006
Merck Generics	Mylan NV	6.62	12 May 2007	2 October 2007
Hexal AG	Novartis AG	5.69	21 February 2005	7 June 2005
Actavis Group HF	Allergan plc	5.61	25 April 2012	31 October 2012
Developed markets branded generics pharmaceuticals	Mylan NV	5.61	14 July 2014	27 February 2015

^a^Data were gathered from Bloomberg L.P. Finance

^b^Included completed deals with a generic target

^c^Financial figures are in billions of United States dollars not adjusted for inflation and rounded to two decimal places

## Discussion

Prior to the year 2000, mergers and acquisitions played a minimal role in the generic drug sector. The growth in mergers and acquisitions afterward is consistent with industry reports noting that generic firms have expanded their operations through mergers and acquisitions in recent years [1, 18]. Our analysis confirms that 2015 and 2016 in particular experienced record levels of merger and acquisition activity in the generic drug industry, based on the date of deal completion. Our results suggest that there is a substantial movement in the last two years in the generic sector towards using merger and acquisition deals to grow rather than traditional greenfield investments. The identification of the largest mergers and acquisitions show that since 2011, three acquisitions were announced at more than $10 billion, suggesting that larger companies are being acquired.

The literature provides some explanations for the recent proliferation of merger and acquisition deals in the pharmaceutical industry [1, 4–6]; they include: 1 – generic firms can benefit from economies of scale through savings on administrative and capital costs; 2 – the loss of major patents have brought brand-name manufacturers to enter the generic drug market; 3 – mergers and acquisitions may be pursued as a way to enter emerging markets faster; 4 – loans can be obtained easily due to low-interest rates and because banks consider the healthcare sector as more reliable; 5 – investors’ pressure to create shareholder value and pressure; 6 – fear of ‘missing out’, where all the good assets are being bought out; 7 – vertical integration to buy parts or sections of the supply chain; 8 – the emergence of biosimilars also created new market niches for more complex products with better market exclusivity, thereby creating more appealing targets for mergers and acquisitions (in the United States, new biosimilars are granted 12 years of market exclusivity, compared to 180 days given to traditional generic drugs entering the market).

With the recent wave of mergers and acquisitions, it appears that the industry is consolidating; however, the number of enterprises in this industry grew from 2010 to 2015 [1]. Despite the appearance of consolidation, the top four generic pharmaceutical manufacturing firms in the United States made only 26.4% of the industry’s total revenue in 2015 [1]. The largest generic company, Pfizer Global Established Products, represented 9% of the global market value for generics and the top ten global companies represented less than 40% of global market value [19]. The Herfindahl-Hirschman index (HHI), a commonly accepted measure of market concentration, was estimated at 0.021 for the global generic sector in February 2016, way below the United States Department of Justice threshold of 0.25 where caution starts to be exercised by antitrust authorities [19]. However, the low overall concentration ratio might be misleading as compared to concentration index for specific therapeutic categories or molecules. For instance, a study that analyzed 1200 generic drugs showed that nearly half of the drugs had an HHI value exceeding 0.5, which is considered duopoly like competition level [17]. The study also showed that increases in generic drug prices in the United States are strongly related with market competition levels. In fact, several companies developed a novel business model based on the domination on non-competitive markets for older drugs by cornering niche generic markets through mergers and acquisitions in order to substantially increase prices [20]. Mergers and acquisitions were thus an important factor to explain the large price increases for different generics like albendazole (treatment for intestinal parasites), dextroamphetamine (treatment for attention-deficit disorder), and pyrimethamine (treatment for toxoplamosis), nitroprusside (treatment for high blood pressure) and isoprotenerol (used in cardiac emergencies) [11, 13, 20].

Our results show that merger and acquisition activity among generic drug companies have become a major trend in this industry since 2004, but that this trend has accelerated in 2015 and 2016, and that the United States target companies have been the center of this acceleration. It is important to note that the record level of mergers and acquisitions in the United States appeared after two years of price increases for a large proportion of generics in the country [3]. Mergers and acquisitions should not be considered the root cause of generic drug price increases, but rather as a factor exacerbating the trend of rising prices. The results also show that the focus on United States generic targets in the last few years is not linked to the need to enter emerging markets or vertical integration with manufacturers of raw ingredients in emerging countries. The significant price increase for many generic drug products in the United States also means that mergers and acquisitions are not simply a means to increase efficiency and reduce costs.

While there was a “patent cliff” starting in 2010 when many blockbuster brand-name products lost their patent protection, greatly increasing the market for generics, many governments also imposed measures like generic substitution, heightening the demand for generics [15–19, 21]. Governments and private drug plans have also imposed downward pressure on the price of generic drugs, forcing a restructuring in some parts of the industry [22]. This downward price pressure from large buyers is problematic for smaller manufacturers, creating incentives for mergers and acquisitions among sellers to adjust to the market factors [23].

Typically, mergers and acquisitions reduce the number of manufacturers for specific drugs in some therapeutic niches, which can potentially lead to price increases. Mergers and acquisitions are also identified as a potential cause for drug shortages since they may result in supply disruptions based on business decisions to narrow the focus of the product line, discontinue products or to shift manufacturing to another facility [16]. For example, after acquiring the largest producer of generic injectable drugs in 2015, Hospira, Pfizer became the only supplier of injectable sodium bicarbonate in Canada [24]. The drug, similar to baking soda, is widely used for emergency procedures, open heart surgery or during some chemotherapy treatment. After manufacturing problems, and because of a lack of alternative manufacturers, Canada had a shortage of the drug in 2017 [24].

The record level of mergers and acquisitions in the last two years indicate that the economic structures of the generic sector are shifting, especially in the United States. In this context of increasing economic restructuring, countries must adapt their regulations and procurement policies accordingly in order to protect themselves against abusive price increases or drug shortages. The market forces in the generic sector do not necessarily ensure lower prices and safe supply for all generics. Governments must thus develop institutional capacities to deal with potential problems.

Governments should consider implementing for their public drug plan a procurement process with tenders that include specific clauses to ensure the safety of the drug supply and reduce drug shortages [25, 26]. The establishment of a public generic manufacturer, like what is found in Sweden, could also be explored as a way to deter predatory pricing and reduce drug shortages [15, 24, 25]. Antitrust authorities should also examine the current practices of generic manufacturers in this context of merger mania.

In particular, in the United States, the antitrust laws protect consumers only against anticompetitive strategies such as price fixing among competitors. Generic manufacturers that legally obtain a monopoly on a product through mergers and acquisitions are free to unilaterally increase prices [11]. To ensure more market competition between manufacturers, the United States Food and Drug Administration could create special pathways for foreign manufacturers or new competitors to promote competition and allow the market to work more efficiently [11]. Because of the magnitude of current mergers and acquisitions in an evolving generic sector, solely relying on market forces might make some essential generic drugs inaccessible for many due to high costs or shortages.

### Limitations

Although this study starts to address the lack of information on mergers and acquisitions in the generic drug industry, it has several limitations. The most noteworthy limitation of this study is that we did not directly associate merger and acquisition deals with their specific outcomes such as the price or shortages of the products involved in the mergers. Looking at the years 2015 and 2016, it is too early to reliably determine the impacts of the mergers and acquisitions. Another limitation was that the announced value was “not announced” in cases where a private company acquired the generic drug company; the likely result is the underestimation of the value of mergers and acquisitions.

## Conclusions

This analysis traced merger and acquisition activity in the generic drug sector from 1995, when deals were negligible, to 2016 when the total value of deals rose to $44 billion, representing 35% of all mergers and acquisitions among pharmaceuticals. The surge in mergers and acquisitions recorded for 2015 and 2016 indicates sectorial transformations, for which further research will be needed to determine the specific impacts on the availability and pricing of generic drugs. We can only observe that the structures of the generic market are changing. The generic drug industry has changed since 1995, and it is thus necessary to update regulations and procurement policies accordingly to develop institutional capacities to deal with potential problems. In particular, Antitrust authorities should scrutinize current practices, public drug plans should consider modifying their procurement process to ensure the safety of drug supply, and governments could also explore the possibility of establishing public generic manufacturers.

## Additional files

Additional file 1: Global, United States, and Global excluding the United States deal specific data. (PDF 313 KB)
