# Supplementary material for: Merger mania: mergers and acquisitions in the generic drug sector from 1995 to 2016
Source: Global Health. 2017 Aug 22;13:62. doi: 10.1186/s12992-017-0285-x (PMC5567637; doi:10.1186/s12992-017-0285-x)
Supplement: Supplementary file 1 — Global, United States, and Global excluding the United States deal specific data. (PDF 313 KB) [file 12992_2017_285_MOESM1_ESM.zip › 12992_2017_285_MOESM1_ESM/Deal specific data Global.pdf]

Completed-Year	Announce Date	Target Name	Acquirer Name	Seller Name	Announced Total Value (mil.)	Payment Type	TV/EBITDA	Deal Status
2016	2015-07-27	Generic drug business	Teva Pharmaceutical Industries Ltd	Allergan plc	39564	Cash and Stock		Completed
2016	2016-05-13	Generics Business/Illinois	Mylan NV	Renaissance Acquisition Holdings LLC	950	Cash		Completed
2016	2015-07-23	Gavis Pharmaceuticals LLC,Novel Laboratories Inc	Lupin Ltd		880	Undisclosed		Completed
2016	2016-06-28	Generic drugs portfolio	Mayne Pharma Group Ltd	Teva Pharmaceutical Industries Ltd,Allergan pl	652	Cash		Completed
2016	2016-03-29	Epic Pharma LLC,Epic RE Holdco LLC	Humanwell Healthcare Group Co Ltd		550	Cash		Completed
2016	2015-09-04	InvaGen Pharmaceuticals Inc,Exelan Pharmaceuticals Inc	Cipla Ltd/India		550	Cash		Completed
2016	2016-06-11	Portfolio of 8 abbreviated new drug applications	Dr Reddy's Laboratories Ltd	Teva Pharmaceutical Industries Ltd,Allergan pl	350	Cash		Completed
2016	2016-07-25	Acino AG,Acino Supply AG	Luye Pharma Group Ltd	Acino International AG	269.18	Cash		Completed
2016	2016-01-07	Avioq Inc	Shandong Oriental Ocean Sci-Tech Co Ltd		68.22	Cash		Completed
2016	2016-01-15	Huainan Chaoyang Hospital Management Co Ltd	Guizhou Yibai Pharmaceutical Co Ltd		53.24	Cash		Completed
2016	2015-09-08	EIMC United Pharmaceuticals	Hikma Pharmaceuticals PLC		38.33	Cash		Completed
2016	2015-09-19	Ranbaxy's Solus & Solus Care divisions	Strides Shasun Ltd	Sun Pharmaceutical Industries Ltd	25.09	Cash		Completed
2016	2016-05-03	KYNAMRO develop & commercialize rights	Kastle Therapeutics LLC	Ionis Pharmaceuticals Inc	25	Cash and Stock		Completed
2016	2016-02-08	Generic Partners Holdings Co Pty Ltd	Strides Shasun Ltd		10.65	Cash		Completed
2016	2016-04-27	Biozone Laboratories Inc	Flavor Producers Inc	MusclePharm Corp	8.3	Cash		Completed
2016	2016-04-21	Celtis Pharm Co Ltd	TELCON Inc		6.15	Cash		Completed
2016	2016-11-24	PediaCare brand/Moberg Pharma AB	Strides Shasun Ltd	Moberg Pharma AB	5.6	Cash		Completed
2016	2016-12-30	Hunan Fangsheng BioPharma Inc	Hainan Boda Pharmaceutical Co Ltd	Hunan Fangsheng Pharmaceutical Co Ltd	2.19	Cash		Completed
2016	2016-10-26	Research & development facility/Belgium	VolitionRX Ltd		1.32	Cash		Completed
2016	2016-02-24	ADASUVE US Commerical Rights	Alexza Pharmaceuticals Inc	Teva Pharmaceutical Industries Ltd	0.67	Stock		Completed
2016	2016-02-11	Beyond Human LLC	Innovus Pharmaceuticals Inc		0.63	Cash		Completed
2016	2016-03-31	Schutz Dishman Biotech Ltd	Dishman Pharmaceuticals & Chemicals Ltd	Schutz & Co Beteiligungsgesellschaft mbH	0.34	Undisclosed		Completed
2016	2016-05-17	L'Estoig Farmaceutic SL	Graficas Maculart SA		N/A	Undisclosed		Completed
2016	2016-06-24	Smith Brothers Co/The	GR Lane Holdings Ltd		N/A	Undisclosed		Completed
2016	2016-06-28	MENA Operations,MOVICOL & MOVIPREP & KLEAN-PREP & NORMACOL	Acino International AG	Norgine Europe BV	N/A	Cash		Completed
2016	2016-06-06	Confab Laboratories Inc	Mylan NV	RoundTable Healthcare Partners	N/A	Cash		Completed
2016	2016-07-20	3 pre-clinical development programs	Chronos Therapeutics Ltd	Shire PLC	N/A	Cash		Completed
2016	2016-08-01	Tsurukame Chozai Yakkyoku YK	Medical Ikkou Co Ltd		N/A	Undisclosed		Completed
2016	2016-08-02	Schuetz GmbH & Co KG	Biesterfeld AG		N/A	Undisclosed		Completed
2016	2016-06-20	US VMS Business	International Vitamin Corp	Perrigo Co PLC	N/A	Cash		Completed
2016	2016-07-26	Ibudtender LLC	Cannabis Sativa Inc		N/A	Undisclosed		Completed
2016	2016-09-02	Rights for myeloma drug	Amgen Inc	Boehringer AG	N/A	Cash		Completed
2016	2016-10-06	Convey Health Solutions Holdings LLC	New Mountain Capital LLC	ComVest Group Inc	N/A	Cash		Completed
2016	2016-06-16	Certain European rights to lithium based products	Teofarma Srl	GlaxoSmithKline PLC	N/A	Cash		Completed
2016	2016-08-08	Marifarm Proizvodnja In Storitve Doo	Arterium Korporatsiya		N/A	Undisclosed		Completed
2016	2015-10-21	7 brands/Johnson & Johnson	Strides Shasun Ltd	Johnson & Johnson	N/A	Cash		Completed
2016	2016-10-13	Brolene Eye Drops	Adcock Ingram Holdings Ltd	Genop Healthcare Pty Ltd	N/A	Cash		Completed
2016	2016-10-25	Pharmalys Laboratories SA,Pharmalys Tunisia Ltd SA,Pharmalys Africa Sarl	HOCHDORF Holding AG		N/A	Undisclosed		Completed
2016	2016-12-28	6 Brands/MSD	Cadila Healthcare Ltd		N/A	Cash		Completed
2016	2016-09-28	Francois Hyafil research centre/France	Oncodesign	GlaxoSmithKline PLC	N/A	Cash		Completed
2016	2016-12-29	ept pregnancy test kit brand	NFI Products Inc	Prestige Brands Holdings Inc	N/A	Cash		Completed
2016	2016-04-08	Alkion Biopharma SAS	Evonik Industries AG		N/A	Undisclosed		Completed
2015	2015-02-05	Hospira Inc	Pfizer Inc		16807.25	Cash	23.2	Completed
2015	2015-05-18	Par Pharmaceutical Holdings Inc	Endo International PLC	Partners VI LP),TPG Capital Management LP	8090.04	Cash and Stock	32.49	Completed
2015	2014-07-14	Developed markets branded generics pharmaceuticals	Mylan NV	Abbott Laboratories	5609.97	Stock		Completed
2015	2014-10-09	Lineage Therapeutics Inc,Tower Holdings Inc	Impax Laboratories Inc	Table Healthcare Partners (Fund: Roundtable H	691.3	Cash		Completed
2015	2015-02-05	Branded respiratory business in the US and Canada	AstraZeneca PLC	Allergan plc	600	Cash		Completed
2015	2015-08-24	Talazoparib Drug Rights	Medivation Inc	BioMarin Pharmaceutical Inc	410	Cash		Completed
2015	2015-05-14	Pharmstandard PJSC	Augment Investments Ltd		392.73	Cash		Completed
2015	2015-05-21	Generic pharmaceutical business & certain assets	Strides Shasun Ltd	Aspen Pharmacare Holdings Ltd	299.71	Cash		Completed
2015	2014-08-20	Gansu Chengji Bio Pharmaceutical Co Ltd	Hybio Pharmaceutical Co Ltd		214.98	Cash and Stock		Completed
2015	2015-02-24	Neutec Toplam Kalite Yonetimi Sanayi Ticaret AS	Takeda Pharmaceutical Co Ltd	Neutec Ilac Sanayi Ve Ticaret AS	121.26	Cash		Completed
2015	2015-01-27	Polytech-Domilens GmbH	Stirling Square Capital Partners LLP		107.7	Cash		Completed
2015	2015-07-17	Beijing Jiu He Pharmaceutical Ltd	CHINAGRANDPHARM	ngbo CDH Jinxiu Investment Management Co I	72.8	Cash		Completed
2015	2014-10-15	Litha Healthcare Group Ltd	Endo International PLC		41.59	Cash	11.57	Completed
2015	2015-05-12	Pantheon's Mexican operations	Perrigo Co PLC	Patheon Inc	34	Cash		Completed
2015	2015-07-07	Guangzhou Pharmaceutical Research Institute Co Ltd	BAIYUNSHAN PH	Guangzhou Pharmaceutical Holdings Ltd	25.79	Cash		Completed

2015	2015-07-13	22 generic drug products	ANI Pharmaceuticals Inc	Teva Pharmaceutical Industries Ltd	25	Cash		Completed
2015	2015-03-17	4 targeted oncology development programs worldwide	Ignyta Inc	Teva Pharmaceutical Industries Ltd	11.44	Stock		Completed
2015	2015-04-09	CynoGen Inc	Rosetta Genomics Ltd	Prelude Corp	3.51	Cash and Stock		Completed
2015	2015-08-12	Semi-occlusive wound dressing	Madison Ventures Inc	Ocure Ltd	0.28	Cash		Completed
2015	2015-03-30	Domestic Generic Pharmaceuticals Business	Amneal Pharmaceuticals LLC	Allergan plc	N/A	Undisclosed		Completed
2015	2015-06-30	Time-Cap Laboratories Inc	Marksans Pharma Ltd		N/A	Undisclosed		Completed
2015	2015-07-20	OmniLytics Inc	Phagelux Inc		N/A	Undisclosed		Completed
2015	2015-02-12	PDX business unit	Crown Bioscience Inc	Molecular Response LLC	N/A	Cash		Completed
2015	2015-04-10	Aurobindo Pharma Australia Pty Ltd	Allergan plc	Aurobindo Pharma Ltd	N/A	Undisclosed		Completed
2015	2015-02-10	RAK Pharmaceuticals Pvt Ltd	Gulf Pharmaceutical Industries PSC	RAK Ceramics Bangladesh Ltd	N/A	Undisclosed		Completed
2015	2015-10-28	Acris Antibodies GmbH	OriGene Technologies Inc		N/A	Undisclosed		Completed
2015	2015-12-03	MSD's Portfolio of Glaucoma Products	Mundipharma Ophthalmology Products Ltd	Merck & Co Inc	N/A	Cash		Completed
2015	2015-12-14	Bryan Ohio unit/Sun Pharmaceutical Industries Ltd	Mylan NV	Sun Pharmaceutical Industries Ltd	N/A	Cash		Completed
2015	2015-06-22	Alvogen Inc	Pte Ltd,CVC Capital Partners Ltd,Vatera Healt	Pamplona Capital Management LLP	N/A	Cash		Completed
2015	2015-10-14	Primm Pharma SRL	Xbrane Bioscience AB		N/A	Undisclosed		Completed
2015	2015-09-16	Medicamen Biotech Ltd	Shivalik Rasayan Ltd		N/A	Cash		Completed
2015	2015-03-02	Sellersville facility	G&W Laboratories Inc	Teva Pharmaceutical Industries Ltd	N/A	Cash		Completed
2015	2015-11-25	Groupe LCD SRL	Biogroup SELAFA		N/A	Undisclosed		Completed
2015	2015-11-09	International Pharmaceutical Generics Ltd	Emcure Pharmaceuticals USA Inc		N/A	Undisclosed		Completed
2014	2013-10-02	Acino International AG	c Capital Fund VII LP),Avista Capital Holdings LP (Fund: Avista Capital Partners III LP)		577.95	Cash	12.39	Completed
2014	2014-06-24	DAVA Pharmaceuticals Inc	Endo International PLC		575	Cash		Completed
2014	2013-08-28	Boca Pharmacal Inc	Endo International PLC		225	Cash		Completed
2014	2014-01-16	Alvogen Korea Ltd/Old	Lotus Pharmaceutical Co Ltd	Alvogen Asia Pacific Holdings Ltd	157.24	Cash		Completed
2014	2014-04-01	Silom Medical International Co Ltd	Allergan plc		100	Cash		Completed
2014	2014-03-27	PACK Pharmaceuticals LLC	Aceto Corp		85	Cash and Stock		Completed
2014	2014-12-08	Oriola-KD's Russian businesses	Apteki 366 OOO	Oriola-KD OYJ	68.73	Cash		Completed
2014	2014-02-28	Australia OTC products	Perrigo Co PLC	Aspen Global Inc	51	Cash		Completed
2014	2014-07-21	India branded generics business	Strides Shasun Ltd	Bafna Pharmaceuticals Ltd	7.98	Cash		Completed
2014	2014-06-03	KPX Bio Tech Co Ltd	Sungwun Pharmacopia Co Ltd	Private Investor,KPX Holdings Corp	5.98	Cash		Completed
2014	2014-05-15	OrganiGram Inc	OrganiGram Holdings Inc		3.28	Stock		Completed
2014	2014-10-06	Vida Laboratories Ltd	Private Investor	Silk Road Energy Services Group Ltd	2.06	Cash		Completed
2014	2014-07-18	Quantum Healthcare Thailand Co Ltd,Oncology Imaging Systems Korea Co Ltd	Quantum Energy Ltd		N/A	Undisclosed		Completed
2014	2014-02-03	Nanomi BV	Lupin Ltd		N/A	Undisclosed		Completed
2014	2014-04-02	Alvogen Inc	Pamplona Capital Management LLP		N/A	Cash		Completed
2014	2014-04-17	Four branded ophthalmic and topical products	Allergan plc	Akorn Inc	N/A	Cash		Completed
2014	2014-04-16	Antioxidant ibuprofen and propofol businesses	SI Group Inc	Albemarle Corp	N/A	Cash		Completed
2014	2014-12-03	WindStar Medical GmbH	HQ Capital Private Equity LLC		N/A	Cash		Completed
2014	2014-05-01	Otaniguchi Pharmacy Business Units	Weeds Co Ltd	Otaniguchi Pharmacy KK	N/A	Undisclosed		Completed
2014	2014-01-13	CGRP antibody	Eli Lilly & Co	Arteaus Therapeutics LLC	N/A	Cash		Completed
2014	2014-11-20	Huvepharma EOOD	Advance Properties Ood	TRG Management LP	N/A	Cash		Completed
2014	2014-06-19	Sunsho Pharmaceutical Co Ltd	Carlyle Group LP/The		N/A	Undisclosed		Completed
2013	2013-08-13	Bever Pharmaceutical Pte Ltd	Pharmstandard PJSC		590	Cash and Stock		Completed
2013	2013-03-11	Simcere Pharmaceutical Group	Right Lane Ltd,King View Development International Ltd,New Good Management Ltd,Assure		114.56	Cash	11.98	Completed
2013	2011-08-30	Sanitas AB	Valeant Pharmaceuticals International Inc		57.76	Cash	12.94	Completed
2013	2013-05-01	Metronidazole 1.3% Vaginal Gel	Allergan plc	Valeant Pharmaceuticals International Inc	55	Cash		Completed
2013	2013-06-04	Novavax AB	Novavax Inc		26.48	Stock		Completed
2013	2013-12-26	31 generic drug products	ANI Pharmaceuticals Inc	Teva Pharmaceutical Industries Ltd	12.5	Cash		Completed
2013	2013-04-30	Pitney Pharmaceuticals Pty Ltd	PharmAust Ltd		4.55	Stock		Completed
2013	2013-08-29	Beijing Lingrui Sanity Material Co Ltd	Henan Lingrui Group Co Ltd	Henan Lingrui Pharmaceutical Co	3.1	Cash		Completed
2013	2013-02-13	OTC Pharmaceutical assets	Valeant Pharmaceuticals International Inc	Lek-Am Sp zoo	N/A	Cash		Completed
2013	2013-01-15	Labormed-Pharma SA	Alvogen Inc	Advent International Corp	N/A	Undisclosed		Completed
2013	2013-07-02	Libertas Pharma Inc	Mayne Pharma Group Ltd		N/A	Undisclosed		Completed
2013	2013-07-24	Opalia Pharma	Recordati SpA		N/A	Cash		Completed
2013	2013-09-30	4 generic medical products	Amneal Pharmaceuticals LLC	Allergan plc	N/A	Cash		Completed
2013	2013-10-23	CBD Life Sciences Inc	GelStat Corp		N/A	Undisclosed		Completed
2013	2013-11-01	CALADRYL	Piramal Enterprises Ltd	Valeant Pharmaceuticals International Inc	N/A	Cash		Completed
2013	2013-11-07	Naprelan rights	Alvogen Inc	Shionogi & Co Ltd	N/A	Cash		Completed
2013	2013-12-16	Jubilant Cadista Pharmaceuticals Inc,Vertical Pharmaceuticals Inc	Avista Capital Holdings LP		N/A	Undisclosed		Completed
2013	2013-12-23	Sogeval SA	Ceva Sante Animale SA	Sofiproteol	N/A	Undisclosed		Completed
2013	2013-11-04	Opalia Pharma	Recordati SpA	Abraaj Group Ltd/The	N/A	Cash		Completed
2013	2013-12-16	ZellBios SA	DPE Deutsche Private Equity GmbH	Ergon Capital Partners SA	N/A	Cash		Completed
2012	2012-04-25	Actavis Group HF	Allergan plc	Novator EHF	5610.42	Cash		Completed

2012	2012-07-16	Par Pharmaceutical Cos Inc	Partners VI LP),TPG Capital Management LP (Fund: TPG Biotechnology Partners IV LP)		1934.2	Cash	9.86	Completed
2012	2012-10-29	Banner Pharmacaps Inc	Patheon Inc	Vion NV	255	Cash		Completed
2012	2012-02-21	Pharmaplan Pty Ltd	Litha Healthcare Group Ltd	Paladin Labs Inc	78.85	Cash and Stock		Completed
2012	2012-12-28	Cobrek Pharmaceuticals Inc	Perrigo Co PLC		45	Cash		Completed
2012	2011-05-23	Par Formulations Pvt Ltd	Par Pharmaceutical Cos Inc		37.6	Cash		Completed
2012	2012-02-24	Suzhou First Pharmaceutical Co Ltd	China NT Pharma Group Co Ltd	Suzhou Pharmaceutical Group Co Ltd	9.53	Cash		Completed
2012	2012-07-10	Anhui Wei Na Shengmingkexue Jishu Kai Fa	Daito Pharmaceutical Co Ltd		7	Cash		Completed
2012	2012-04-18	Certain Assets	Valeant Pharmaceuticals International Inc	Atlantis SA de CV	5.4	Cash		Completed
2012	2012-01-17	Oncogenerix Inc	Midatech Pharma US Inc		1.45	Stock		Completed
2012	2012-10-02	Macleods Pharmaceuticals Ltd	Neogen Corp		N/A	Undisclosed		Completed
2012	2012-05-29	Exclusive Rights to Intellectual Property	Raptor Pharmaceutical Corp	McGill University	N/A	Undisclosed		Completed
2012	2012-07-04	YPA KK	Kamei Corp		N/A	Undisclosed		Completed
2012	2012-02-21	Pharmaplan Pty Ltd	Paladin Labs Inc		N/A	Undisclosed		Completed
2012	2012-07-03	WISAP Medical Technology GmbH	Blue Cap AG		N/A	Cash		Completed
2011	2011-05-19	Nycomed A/S	Takeda Pharmaceutical Co Ltd	ordic Capital Fund V LP),Avista Capital Holding	13732.8	Cash		Completed
2011	2011-01-20	Substantially all assets	Perrigo Co PLC	Paddock Laboratories Inc	540	Cash		Completed
2011	2011-05-24	Sanitas AB	Valeant Pharmaceuticals International Inc	ternational (Fund: TRG Growth Partnership LP	463.05	Cash	11.86	Completed
2011	2011-08-24	Anchen Pharmaceuticals Inc	Par Pharmaceutical Cos Inc		410	Cash		Completed
2011	2011-09-26	Teva-Kowa Pharma Co	Teva Pharmaceutical Industries Ltd	Kowa Co Ltd/Aichi	150	Cash		Completed
2011	2011-08-01	C&O Pharmaceutical Technology Holdings Ltd	Shionogi & Co Ltd		128.58	Cash	8.63	Completed
2011	2011-07-07	Biozone Laboratories Inc,Equachem LLC,Equalan Pharmaceuticals LLC	Cocrystal Pharma Inc		104.98	Stock		Completed
2011	2010-12-06	Caraco Pharmaceutical Laboratories Ltd	Sun Pharmaceutical Industries Ltd		46.8	Cash		Completed
2011	2011-01-25	Tenth of Ramadan Pharmaceuticals and Diagnostic Re	Sphinx Private Equity Management (Fund: Sphinx Turnaround Fund),Compass Capital		40	Cash		Completed
2011	2011-05-15	Land & Building	Unnamed Buyer	Allergan plc	4.7	Cash		Completed
2011	2011-06-01	Flower Pharmacy Tamashiro	Medical Ikkou Co Ltd		N/A	Cash		Completed
2011	2011-06-15	Home Wellness Inc	Riverside Co/The		N/A	Cash		Completed
2011	2011-06-17	China Resources Purenhong Beijing Pharmaceutical C	a Resources Pharmaceutical Commercial Group Co		N/A	Undisclosed		Completed
2011	2011-06-06	NextEra As	AS (Fund: Birk Venture/Fund),Olsen Capital & Consulting AS		N/A	Cash		Completed
2011	2011-07-08	Certain Assets	CH Boehringer Sohn AG & Co KG	Svanova Biotech AB	N/A	Cash		Completed
2011	2011-08-02	Certain Assets	SOHM Inc	Novatrend Medicament Pvt Ltd	N/A	Cash		Completed
2011	2011-09-01	Clover YK	Medical Ikkou Co Ltd		N/A	Undisclosed		Completed
2011	2011-09-01	Detach N' Go	Apex Medical Corp/United States	Inventures Products LLC	N/A	Cash		Completed
2011	2011-01-19	Biolek	Pharmstandard PJSC		N/A	Cash		Completed
2011	2011-03-25	Exclusive license	XTL Biopharmaceuticals Ltd	MinoGuard	N/A	Cash		Completed
2011	2011-11-15	ADDvance Brand	Adcock Ingram Holdings Ltd	Peppina Sales	N/A	Cash		Completed
2011	2011-01-19	Biolek	Farmstandart OAO		N/A	Undisclosed		Completed
2011	2011-10-17	Square Cephalosporins Ltd	Square Pharmaceuticals Ltd		N/A	Undisclosed		Completed
2010	2010-09-28	Qualitest and Vintage Pharmaceuticals	Endo International PLC	Apax Partners LLP	1200	Cash		Completed
2010	2009-11-05	Swedish Orphan International AB	Swedish Orphan Biovitrum AB		500.25	Cash		Completed
2010	2010-03-04	CRINONE Progesterone Gel Product Line	Allergan plc	Juniper Pharmaceuticals Inc	47	Cash		Completed
2010	2010-10-28	ANB Laboratories Co Ltd	Bangkok Dusit Medical Services PCL		24.41	Cash		Completed
2010	2009-11-18	Israel Cosmetics Business	Sano-Brunos Enterprises Ltd	Emilia Development Ltd	22.56	Cash		Completed
2010	2010-05-11	Nihon Pharmaceutical Industry Co Ltd	Nippon Chemiphar Co Ltd		12.4	Stock		Completed
2010	2010-08-06	Apredica LLC	Cyprotex PLC		5.87	Cash and Stock		Completed
2010	2008-06-30	Taro Pharmaceutical Industries Ltd	Sun Pharmaceutical Industries Ltd		0.2	Cash		Completed
2010	2010-05-05	Certain Assets	Perrigo Co PLC		N/A	Cash		Completed
2010	2010-05-07	Manufacturing Facilities at Kurkumbh	Cipla Ltd/India		N/A	Cash		Completed
2010	2010-05-26	Intellectual Ppty & Assets	Perrigo Co PLC	Novel Laboratories Inc	N/A	Cash		Completed
2010	2010-06-18	Medisa Shinyaku Inc	Sawai Pharmaceutical Co Ltd	Sumitomo Corp	N/A	Undisclosed		Completed
2010	2010-08-03	Pharmaceutical Products	AA Pharma	Apotex Inc	N/A	Cash		Completed
2010	2010-09-02	Vindexpharm ZAO	Pharmstandard PJSC		N/A	Cash		Completed
2010	2010-09-30	Metoprolol Succinate Product	Intas Pharmaceuticals Ltd	Orbus Pharma Inc	N/A	Cash		Completed
2010	2010-09-30	Daichi Kasei Co Ltd	Towa Pharmaceutical Co Ltd	Private Investor,KNC Laboratories Co Ltd	N/A	Undisclosed		Completed
2010	2010-12-23	Confab Laboratories Inc	RoundTable Healthcare Partners		N/A	Cash		Completed
2010	2010-12-07	Biotec Services International Ltd	p,Finance Wales Investments Ltd (Fund: Wales JEREMIE Fund)		N/A	Cash		Completed
2009	2008-06-18	Zentiva NV	Sanofi		2943.38	Cash	14.39	Completed
2009	2009-01-26	Farma APS	oup,Magnum Industrial Partners SL (Fund: Magnum Capital LP)		236.63	Cash		Completed
2009	2008-10-16	Laboratories Teva Ltd Israel,Abic Veterinary Products Ltd Israel,Assia Pharmaceut	Phibro Animal Health Corp	Teva Pharmaceutical Industries Ltd	47	Cash		Completed
2009	2009-12-30	Immunocorp Consumer Health AS	Sana Pharma AS	Biotec Pharmacon ASA	5.43	Cash		Completed
2009	2009-01-28	RxElite Holdings Inc	Piramal Enterprises Ltd	RXElite Inc	4.2	Cash		Completed
2009	2009-08-28	Jiangxi Hangtian Tailishi Pharmaceutical Co Ltd	Renhe Pharmacy Co Ltd	estor,Nanchang Hangtian Investment Developr	2.64	Cash		Completed
2009	2009-03-31	Beijing Univision Pharmaceutical Co Ltd	Center Laboratories Inc		0.1	Cash		Completed

2009	2009-04-02	Laboratorios Kendrick SA	Sanofi		N/A	Undisclosed		Completed
2009	2009-02-25	INVO BioScience Inc/Old	INVO BioScience Inc		N/A	Stock		Completed
2009	2009-04-01	Certain Assets	Par Pharmaceutical Cos Inc	Marina Biotech Inc	N/A	Cash		Completed
2009	2009-03-19	si Saglik Urunleri Sanayi ve Ticaret AS,EOS Eczacibasi Ozgun Kimyasal Urunler Sa	Zentiva NV	IS Eczacibasi Ilac ve Sinai ve Finansal Yatirim	N/A	Undisclosed		Completed
2009	2009-08-03	Portfolio of 20 branded generic products	Nycomed SCA SICAR	Sanofi	N/A	Cash		Completed
2009	2009-07-03	Ostelin brands	Sanofi	Reckitt Benckiser Group PLC	N/A	Cash		Completed
2009	2009-11-30	Breckenridge Pharmaceutical Inc	Pensa Pharma SA		N/A	Undisclosed		Completed
2009	2008-12-09	Generis-Farmaceutica SA	Magnum Industrial Partners SL		N/A	Cash		Completed
2009	2009-07-24	Product Portfolio & Ozone Brand & Intellectual Pro	Advent International Corp	Ozone Laboratories Group	N/A	Cash		Completed
2009	2009-02-18	Agennix Inc	GPC Biotech AG		N/A	Stock		Completed
2008	2008-07-18	Barr Pharmaceuticals Inc	Teva Pharmaceutical Industries Ltd		8833.22	Cash and Stock	11.96	Completed
2008	2008-09-11	Zoetis Products LLC	KP Pharmaceuticals LLC		1253.63	Cash		Completed
2008	2008-07-21	Symbion Consumer	Sanofi	Primary Health Care Ltd	545.83	Cash		Completed
2008	2008-02-06	Active Pharmaceutical Ingredients business	3i Group PLC	Zoetis Products LLC	395	Cash		Completed
2008	2007-12-24	Orphan Holdings Pty Ltd	Sigma Pharmaceuticals Ltd		113.26	Cash		Completed
2008	2008-11-25	Generic Pharmaceutical Products	Allergan plc	Teva Pharmaceutical Industries Ltd	36	Cash		Completed
2008	2008-06-04	Oncology Projects	4SC AG	Nordic Capital Svenska AB	21.64	Cash		Completed
2008	2008-04-22	Wuhu Zhong Ren Pharmaceutical Co Ltd	Simcere Pharmaceutical Group		9.27	Cash		Completed
2008	2008-08-25	Immunocorp Animal Health AS	Zilor Group	Biotec Pharmacon ASA	6.99	Cash		Completed
2008	2008-04-16	Nanjing Xinaokang Pharmaceutical Ltd	C&O Pharmaceutical Technology Holdings Ltd		5.72	Cash		Completed
2008	2008-10-20	Lianyungang Kangyuan Pharmacetical Commercial Co L	iangsu Jinglue Enterprise Development Co Lt	Jiangsu Kanion Pharmaceutical Co Ltd	5.29	Cash		Completed
2008	2007-10-03	Zenotech Laboratories Ltd	Ranbaxy Laboratories Ltd		2.56	Cash	123.58	Completed
2008	2007-12-17	Sun Pharmaceutical Sdn Bhd	Sunway Holdings Sdn Bhd		0.75	Cash		Completed
2008	2008-07-10	Sichuan Changao Medicine Co Ltd	C&O Pharmaceutical Technology Holdings Ltd		0.01	Cash		Completed
2008	2008-03-12	Huayuan Pharmaceutical Sale Co Ltd,Shanghai Huayuan Pharmaceutical Technolo	Private Investor	Zhuhai Boyuan Investment Co Ltd	N/A	Cash		Completed
2008	2007-11-15	Eduard Vogt AG	Tentan AG	Galenica AG	N/A	Undisclosed		Completed
2008	2007-12-07	Laboratorio Sanderson SA	Fresenius SE & Co KGaA		N/A	Undisclosed		Completed
2008	2008-04-03	Dr Reddy's Srl	Dr Reddy's Laboratories Ltd		N/A	Undisclosed		Completed
2008	2008-04-17	Altisana UAB	Unnamed Buyer	Sanitas AB	N/A	Undisclosed		Completed
2008	2008-04-24	Tamda SA	Fagron		N/A	Undisclosed		Completed
2008	2008-04-25	Interpharm Holdings Inc	Amneal Pharmaceuticals LLC		N/A	Undisclosed		Completed
2008	2008-05-28	Aquaworx AG	aligna AG		N/A	Cash and Stock		Completed
2008	2008-04-30	Plasmaverarbeitungsgesellschaft	Octapharma AG		N/A	Undisclosed		Completed
2008	2007-03-01	Orphan Pharma International Ltd	EUSA Pharma Ltd		N/A	Undisclosed		Completed
2008	2008-01-03	Certain Assets	Amneal Pharmaceuticals LLC		N/A	Cash		Completed
2008	2008-10-02	Budesonide Formulation	OPKO Health Inc	Teva Pharmaceutical Industries Ltd	N/A	Cash		Completed
2008	2008-12-30	Kadian	Novator EHF	KP Pharmaceuticals LLC	N/A	Cash		Completed
2008	2008-12-23	von der Linde GmbH & Co Immobilienverwaltung KG	Sanacorp Pharmahandel GmbH		N/A	Undisclosed		Completed
2007	2007-05-12	Merck Generics	Mylan NV	Merck KGaA	6620.88	Cash		Completed
2007	2007-05-10	Actavis Group HF	Novator EHF		4542.59	Cash	16.2	Completed
2007	2006-09-21	Hospira Australia Pty Ltd	Hospira Inc		1912.3	Cash	15.89	Completed
2007	2007-11-21	Reliant Pharmaceuticals Inc	GlaxoSmithKline PLC	Bay City Capital LLC	1650	Cash		Completed
2007	2007-03-05	si Saglik Urunleri Sanayi ve Ticaret AS,EOS Eczacibasi Ozgun Kimyasal Urunler Sa	Zentiva NV	IS Eczacibasi Ilac ve Sinai ve Finansal Yatirim	602.14	Cash		Completed
2007	2006-11-08	Plant in Spain	Lonza Group AG	Genentech Inc	191.51	Cash		Completed
2007	2007-11-09	TAD Pharma GmbH	Krka dd Novo mesto	PHW Gruppe Lohman & Co AG	142.28	Cash		Completed
2007	2006-11-30	Abrika Pharmaceuticals Inc	Actavis Group HF		112.67	Cash		Completed
2007	2007-08-31	Forum Bioscience Holdings Ltd	STADA Arzneimittel AG	Ajinomoto Co Inc	76.03	Cash		Completed
2007	2007-09-07	Generics business	Novator EHF	aligna AG	75.08	Cash		Completed
2007	2007-01-09	Natural gas properties/MI	EV Energy Partners LP		71.6	Cash		Completed
2007	2007-03-05	Cerbo Group AB	Nolato AB	Vision Capital Group Ltd	61.44	Cash		Completed
2007	2007-09-24	Pediatric asthma development programs	AstraZeneca PLC	Verus Pharmaceuticals Inc	30	Cash		Completed
2007	2007-09-05	ORCA Pharm GmbH	PLIVA Farmaceutika DD		28.71	Cash		Completed
2007	2007-09-05	ORCA Pharm GmbH	Barr Pharmaceuticals Inc		28.57	Cash		Completed
2007	2007-06-11	Grandix Pharmaceuticals Ltd	Strides Shasun Ltd		24.5	Cash		Completed
2007	2008-01-02	Dr Fisher Farma BV	Mosadex CV	SnowWorld NV	19.74	Cash		Completed
2007	2007-08-28	Biomeda Group	Elder Pharmaceuticals Ltd		6.82	Cash		Completed
2007	2007-06-07	Yangzhou Uni-bio Pharmaceutical Co Ltd	Jinyu Bio-Technology Co Ltd		2.94	Cash		Completed
2007	2007-05-03	Patents	Genovis AB	Biolin Scientific AB	0.3	Cash		Completed
2007	2007-05-02	Pharmatec GmbH	Robert Bosch GmbH	Fresenius SE & Co KGaA	N/A	Undisclosed		Completed
2007	2007-07-09	Wildlife DNA Services Ltd,Food DNA Services Ltd	Hologic Ltd		N/A	Undisclosed		Completed
2007	2007-08-10	Intertech Bio	Breitling Energy Corp		N/A	Stock		Completed
2007	2007-09-17	Qualitest and Vintage Pharmaceuticals	Apax Partners LLP		N/A	Undisclosed		Completed

2007	2007-12-11	Ribbon	Fresenius SE & Co KGaA		N/A	Undisclosed		Completed
2007	2007-04-19	Shenzhou Tongde Pharmaceutical Co Ltd	Zoetis Products LLC		N/A	Undisclosed		Completed
2007	2007-05-09	Zalemark Holding Co Inc	Charis Industries Co		N/A	Undisclosed		Completed
2007	2007-05-22	Rockford-Montgomery Labs Inc	IAHL Corp		N/A	Undisclosed		Completed
2006	2005-07-25	IVAX Corp	Teva Pharmaceutical Industries Ltd		7575.54	Cash or Stock	23.32	Completed
2006	2006-03-28	Sindan	Actavis Group HF		161.23	Cash		Completed
2006	2006-08-14	Adeerall tablets	Barr Pharmaceuticals Inc	Shire PLC	63	Cash		Completed
2006	2006-04-20	Nagoya Plaza Building	Ichigo Real Estate Investment Corp/Old	Taiyo Yakuhin Co Ltd	49.4	Cash		Completed
2006	2006-03-08	Parmed Pharmaceuticals LLC	Cardinal Health Inc	Zoetis Products LLC	40.1	Cash		Completed
2006	2006-02-01	Provalis Healthcare Ltd	Kogen Ltd	Provalis PLC	18.66	Cash		Completed
2006	2005-04-04	Generic pharmaceutical co	Jubilant Life Sciences Ltd		8.25	Cash		Completed
2006	2006-02-27	Shenzhen Liancheng Medicine Co Ltd	C&O Pharmaceutical Technology Holdings Ltd		5.6	Cash		Completed
2006	2006-08-07	PB Diagnostics Ltd	Bio-Rad Laboratories Inc	Provalis PLC	3.05	Cash		Completed
2006	2006-06-23	Patent rights	Sanofi Pasteur Ltd	Provalis PLC	1.25	Cash		Completed
2006	2006-01-11	Nanjing Changao Pharmaceutical Science & Technolog	C&O Pharmaceutical Technology Holdings Ltd		0.09	Cash		Completed
2006	2005-12-15	Cyclacel Ltd	Cyclacel Pharmaceuticals Inc	Cyclacel Group PLC	N/A	Undisclosed		Completed
2006	2006-07-11	Gateway Medical Systems Inc	Ba Research International		N/A	Cash and Stock		Completed
2006	2006-10-16	Blackhawk BioSystems Inc	Bio-Rad Laboratories Inc		N/A	Undisclosed		Completed
2006	2006-12-20	Manufacturing plant	Actavis Group HF	Grandix Pharmaceuticals Ltd	N/A	Cash		Completed
2006	2006-12-25	Shanghai Weike Bio Pharmaceutical Co Ltd	Sanjiu Enterprise Group	henxing Biopharmaceutical and Chemical Co L	N/A	Cash		Completed
2006	2008-01-23	Masterlek	Pharmstandard PJSC		N/A	Undisclosed		Completed
2005	2005-02-21	Hexal AG	Novartis AG		5685.89	Cash		Completed
2005	2005-02-21	Eon Labs Inc	Novartis AG	Santo Holding Deutschland GmbH	1699.23	Cash	13.7	Completed
2005	2005-02-21	Eon Labs Inc	Novartis AG		889.39	Cash	15.03	Completed
2005	2005-10-17	Global generic business	Actavis Group HF	Zoetis Products LLC	810	Cash		Completed
2005	2005-05-20	Actavis Totowa LLC	Actavis Group HF		500	Cash		Completed
2005	2005-02-15	Phoenix Scientific Inc	IVAX Corp		271.85	Cash and Stock		Completed
2005	2005-08-25	Docpharma BVBA	Mylan Laboratories Ltd		263.18	Cash		Completed
2005	2005-04-22	Clifford Hallam Pharmaceuticals Pty Ltd	Joint Venture	Spotless Group Ltd	52.74	Cash		Completed
2005	2004-03-24	Oriental Wave Holding Ltd	Dragon Pharmaceutical Inc		41.75	Undisclosed		Completed
2005	2005-03-07	CIMEX Pharma AG	Acino International AG		26.98	Cash	13.37	Completed
2005	2005-11-04	Apokyn drug rights	Vernalis PLC	Mylan NV	23	Cash		Completed
2005	2005-04-04	Ipsat Therapies Oy	Fund Management Oy,Finnish Industry Investment Ltd,Sitra the Finnish Innovation Fund		8.99	Cash		Completed
2005	2005-06-21	Trace Genetics Inc	DNAPrint Genomics Inc		0.28	Stock		Completed
2005	2005-08-04	CorePharma LLC	RoundTable Healthcare Partners		N/A	Cash		Completed
2005	2005-12-22	8 Products	Par Pharmaceutical Cos Inc		N/A	Cash		Completed
2005	2005-12-23	Able Laboratories Inc	Sun Pharmaceutical Industries Ltd		N/A	Undisclosed		Completed
2005	2005-06-30	AXID Oral Solutions	Braintree Laboratories Inc	Reliant Pharmaceuticals Inc	N/A	Undisclosed		Completed
2005	2005-06-15	Beximco Infusions Ltd	Beximco Pharmaceuticals Ltd		N/A	Stock		Completed
2005	2005-09-30	Keri Pharma Generics	Actavis Group HF	Keri Pharma	N/A	Undisclosed		Completed
2005	2005-01-25	Laboratorios Kendrick SA	Darby Overseas Investments Ltd		N/A	Undisclosed		Completed
2005	2005-09-01	Biotec Pharmacon ASA	Management Group	NorgesInvestor AS	N/A	Undisclosed		Completed
2005	2005-03-10	Nycomed Holding ApS	Nordic Capital Svenska AB	rtners III LP),AlpInvest Partners BV,Blackstone	N/A	Cash		Completed
2004	2004-06-07	Sabex Holdings Ltd	Novartis AG		565.97	Cash		Completed
2004	2004-01-22	Neupogen/Neulasta royalties	Royalty Pharma AG	Memorial Sloan-Kettering Cancer Center	263	Cash		Completed
2004	2004-04-13	Kali Laboratories Inc	Par Pharmaceutical Cos Inc		135	Cash		Completed
2004	2004-12-07	CIMEX Pharma AG	Acino International AG		112.79	Cash and Stock	13.07	Completed
2004	2004-03-09	Convey Health Solutions Holdings LLC	NationsHealth Inc		107.18	Stock		Completed
2004	2004-08-17	Dorom Srl	Teva Pharmaceutical Industries Ltd	Pfizer Inc	85.18	Cash		Completed
2004	2004-10-07	Vetco Inc	Prestige Brands International Inc		49.3	Undisclosed		Completed
2004	2003-09-11	Rights to Loestrin products/US & Canada	Barr Pharmaceuticals Inc	Chilcott UK Ltd	44.93	Cash		Completed
2004	2004-02-04	Caraco Pharmaceutical Laboratories Ltd	Sun Pharmaceutical Industries Ltd		37.57	Cash	17.58	Completed
2004	2004-12-28	Rights to Nordette	Barr Pharmaceuticals Inc	KP Pharmaceuticals LLC	12	Cash		Completed
2004	2004-07-13	Biovena Pharma Sp zoo	Actavis Group HF		8.62	Cash		Completed
2004	2004-07-22	Aquatic animal health operations	Investor Group	Zoetis Products LLC	3.9	Undisclosed		Completed
2004	2004-07-30	Diaclone Research	Hologic Ltd	Orphan Pharma International Ltd	2.85	Undisclosed		Completed
2004	2004-12-23	Riasima Abadi Farma PT	Trimarga Rekatama PT	Indofarma Persero Tbk PT	0.62	Cash		Completed
2004	2004-01-05	Unnamed Target/CN	Bio-One Corp		N/A	Cash and Stock		Completed
2004	2004-01-29	Fenofibrate distribution rights	Reliant Pharmaceuticals Inc	CML HealthCare Inc	N/A	Undisclosed		Completed
2004	2003-12-13	RPG Aventis SA	Ranbaxy Laboratories Ltd	Sanofi-Aventis SA	N/A	Undisclosed		Completed
2004	2004-04-29	Katwijk Farma BV	Apotex Inc	UBS AG	N/A	Undisclosed		Completed
2004	2004-06-30	OTC Portfolio	Lil' Drug Store Products Inc	Juniper Pharmaceuticals Inc	N/A	Undisclosed		Completed

2004	2004-07-19	Linotar & Exorex	Clinuvel Pharmaceuticals Ltd	TransDermal Pharmaceuticals Australia Pty Ltd	N/A	Undisclosed		Completed
2003	2003-04-02	CLL Pharma SA	Neuro Bioscience Inc		43.81	Stock		Completed
2003	2003-04-30	Sanitas AB	Kremi UAB		7.35	Cash		Completed
2003	2003-04-04	Serviphar	Docpharma BVBA		4.13	Cash and Stock		Completed
2003	2003-07-24	Apothecon BV	Docpharma BVBA		1.99	Cash and Stock		Completed
2003	2002-11-26	Interpharm Inc	Interpharm Holdings Inc		N/A	Undisclosed		Completed
2003	2003-04-11	Natural White Inc	Natural White Holdings Ltd		N/A	Undisclosed		Completed
2003	2003-04-10	Accupac Inc	HIG Capital LLC		N/A	Undisclosed		Completed
2003	2003-04-29	Diaclone	Orphan Pharma International Ltd	Biotest AG	N/A	Undisclosed		Completed
2003	2003-10-29	Medicos Laboratories Inc	Inamco International Corp		N/A	Undisclosed		Completed
2003	2003-11-19	LiquiSource Inc	Able Laboratories Inc		N/A	Cash		Completed
2003	2003-02-18	Roscrea Pharmaceutical facility	Taro Pharmaceutical Industries Ltd		N/A	Undisclosed		Completed
2003	2003-03-13	Sea & Ski	Pathfinder Management Inc	Idameneo No.789 Ltd	N/A	Undisclosed		Completed
2002	2002-10-30	Nycomed Holding ApS	rtners III LP),AlpInvest Partners BV,Blackston	Nordic Capital Svenska AB	1125.82	Cash		Completed
2002	2002-03-12	Dr Reddy's Laboratories EU Ltd	Dr Reddy's Laboratories Ltd		12.81	Undisclosed		Completed
2002	2002-02-25	2K Pharmaceuticals AS	PLIVA Farmaceutika DD		3.57	Cash		Completed
2002	2001-06-20	Mohan Medicine Research Institute	Merck KGaA	Kyowa Hakko Kirin Co Ltd	N/A	Undisclosed		Completed
2002	2002-04-02	Laboratoires Merck Sharp & Dohme-Chibret SNC	IVAX Corp	Merck & Co Inc	N/A	Undisclosed		Completed
2002	2002-03-06	GeneThera Inc/Old	GeneThera Inc		N/A	Stock		Completed
2002	2002-08-13	Centex HomeTeam Services lawn care operations	Scotts Miracle-Gro Co/The	Centex LLC	N/A	Undisclosed		Completed
2002	2002-09-03	Eduard Vogt AG	Galenica AG		N/A	Undisclosed		Completed
2001	2001-07-11	Actavis Elizabeth LLC	Zoetis Products LLC	Idameneo No.789 Ltd	660	Cash		Completed
2001	2001-08-24	Nycomed Holding ApS	Nordic Capital Svenska AB	GE Healthcare Ltd	177.48	Cash		Completed
2001	2001-12-18	Reliant Pharmaceuticals Inc	al LLC (Fund: Bay City Capital Fund III LP),Versant Ventures Management LLC (Fund: Versa		150	Cash		Completed
2001	2001-09-12	Aktuapharma SA	Docpharma BVBA		4.39	Cash and Stock		Completed
2001	2000-12-21	Labinca SA	Novartis AG		N/A	Undisclosed		Completed
2001	2000-10-31	Generic Distributors Inc	Unnamed Buyer	Able Laboratories Inc	N/A	Cash		Completed
2001	2001-04-02	Lagap Pharmaceuticals Ltd	Novartis AG	Tiger Brands Ltd	N/A	Undisclosed		Completed
2001	2001-06-27	2 Latvia drug wholesalers	Tamro OYJ		N/A	Undisclosed		Completed
2001	2001-03-29	Rights to certain ANDAs	Zoetis Products LLC	Mylan NV	N/A	Undisclosed		Completed
2000	2000-05-25	Actavis Pharma Inc	Allergan plc		891.44	Stock	14.08	Completed
2000	2000-05-30	Technilab Pharma Inc	Merckle GmbH		51.88	Cash	8.42	Completed
2000	2000-08-04	PDK Labs Inc	PDK Acquisition Corp		19.04	Cash	3.39	Completed
2000	2000-10-13	Laporte Organics Francis SpA	Dipharma SpA	Laporte PLC	14.58	Undisclosed		Completed
2000	2000-09-08	Fagron Group	Omega Pharma NV		N/A	Undisclosed		Completed
1999	1999-05-05	Nycomed Holding ApS	Nordic Capital Svenska AB	GE Healthcare Ltd	450.07	Cash		Completed
1999	1999-08-10	Copley Pharmaceutical Inc	Teva Pharmaceutical Industries Ltd		214.68	Cash	14.12	Completed
1998	1997-06-03	Faulding Inc	Mayne Pharma International Pty Ltd		103.25	Cash		Completed
1997	1996-12-25	Royce Laboratories Inc	Allergan plc		N/A	Stock		Completed
1996	1996-01-29	Biocraft Laboratories Inc	Teva Pharmaceutical Industries Ltd		356.1	Stock		Completed
1995	no deals							

YEAR	ANNOUNCED VALUE (MIL)	ANNOUNCED VALUE (BIL)	# OF DEALS
2016	44010.91	44.01	42
2015	33559.35	33.56	34
2014	1859.22	1.86	22
2013	863.95	0.86	20
2012	7984.45	7.98	15
2011	15620.91	15.62	23
2010	1812.69	1.81	18
2009	3239.38	3.24	17
2008	11229.17	11.23	28
2007	16200.10	16.20	28
2006	7926.17	7.93	17
2005	10273.28	10.27	22
2004	1428.91	1.43	20
2003	57.28	0.06	12
2002	1142.20	1.14	8
2001	991.87	0.99	9
2000	976.94	0.98	5
1999	664.75	0.66	2
1998	103.25	0.10	1
1997	0.00	0.00	1
1996	356.10	0.36	1
1995	0.00	0.00	0
**Total**	**160300.88**	**160.30**	**345**
