# Supplementary material for: Merger mania: mergers and acquisitions in the generic drug sector from 1995 to 2016
Source: Global Health. 2017 Aug 22;13:62. doi: 10.1186/s12992-017-0285-x (PMC5567637; doi:10.1186/s12992-017-0285-x)
Supplement: Supplementary file 1 — Global, United States, and Global excluding the United States deal specific data. (PDF 313 KB) [file 12992_2017_285_MOESM1_ESM.zip › 12992_2017_285_MOESM1_ESM/Deal specific data Non-US.pdf]

Completed-Year	Announce Date	Target Name	Acquirer Name	Seller Name	Announced Total Value (mil.)	Payment Type	TV/EBITDA	Deal Status
2016	2016-06-28	Generic drugs portfolio	Mayne Pharma Group Ltd	Teva Pharmaceutical Industries Ltd,Allergan plc	652	Cash		Completed
2016	2016-07-25	Acino AG,Acino Supply AG	Luye Pharma Group Ltd	Acino International AG	269.18	Cash		Completed
2016	2016-01-15	Huainan Chaoyang Hospital Management Co Ltd	Guizhou Yibai Pharmaceutical Co Ltd		53.24	Cash		Completed
2016	2015-09-08	EIMC United Pharmaceuticals	Hikma Pharmaceuticals PLC		38.33	Cash		Completed
2016	2015-09-19	Ranbaxy's Solus & Solus Care divisions	Strides Shasun Ltd	Sun Pharmaceutical Industries Ltd	25.09	Cash		Completed
2016	2016-02-08	Generic Partners Holdings Co Pty Ltd	Strides Shasun Ltd		10.65	Cash		Completed
2016	2016-04-21	Celtis Pharm Co Ltd	TELCON Inc		6.15	Cash		Completed
2016	2016-11-24	PediaCare brand/Moberg Pharma AB	Strides Shasun Ltd	Moberg Pharma AB	5.6	Cash		Completed
2016	2016-12-30	Hunan Fangsheng BioPharma Inc	Hainan Boda Pharmaceutical Co Ltd	Hunan Fangsheng Pharmaceutical Co Ltd	2.19	Cash		Completed
2016	2016-10-26	Research & development facility/Belgium	VolitionRX Ltd		1.32	Cash		Completed
2016	2016-03-31	Schutz Dishman Biotech Ltd	Dishman Pharmaceuticals & Chemicals Ltd	Schutz & Co Beteiligungsgesellschaft mbH	0.34	Undisclosed		Completed
2016	2016-05-17	L'Estoig Farmaceutic SL	Graficas Maculart SA		N/A	Undisclosed		Completed
2016	2016-06-28	MENA Operations,MOVICOL & MOVIPREP & KLEAN-PREP & NORMACOL	Acino International AG	Norgine Europe BV	N/A	Cash		Completed
2016	2016-06-06	Confab Laboratories Inc	Mylan NV	RoundTable Healthcare Partners	N/A	Cash		Completed
2016	2016-07-20	3 pre-clinical development programs	Chronos Therapeutics Ltd	Shire PLC	N/A	Cash		Completed
2016	2016-08-01	Tsurukame Chozai Yakkyoku YK	Medical Ikkou Co Ltd		N/A	Undisclosed		Completed
2016	2016-08-02	Schuetz GmbH & Co KG	Biesterfeld AG		N/A	Undisclosed		Completed
2016	2016-09-02	Rights for myeloma drug	Amgen Inc	Boehringer AG	N/A	Cash		Completed
2016	2016-06-16	Certain European rights to lithium based products	Teofarma Srl	GlaxoSmithKline PLC	N/A	Cash		Completed
2016	2016-08-08	Marifarm Proizvodnja In Storitve Doo	Arterium Korporatsiya		N/A	Undisclosed		Completed
2016	2016-10-13	Brolene Eye Drops	Adcock Ingram Holdings Ltd	Genop Healthcare Pty Ltd	N/A	Cash		Completed
2016	2016-10-25	Pharmalys Laboratories SA,Pharmalys Tunisia Ltd SA,Pharmalys Africa Sarl	HOCHDORF Holding AG		N/A	Undisclosed		Completed
2016	2016-12-28	6 Brands/MSD	Cadila Healthcare Ltd		N/A	Cash		Completed
2016	2016-09-28	Francois Hyafil research centre/France	Oncodesign	GlaxoSmithKline PLC	N/A	Cash		Completed
2016	2016-04-08	Alkion Biopharma SAS	Evonik Industries AG		N/A	Undisclosed		Completed
2015	2014-07-14	Developed markets branded generics pharmaceuticals	Mylan NV	Abbott Laboratories	5609.97	Stock		Completed
2015	2015-05-14	Pharmstandard PJSC	Augment Investments Ltd		392.73	Cash		Completed
2015	2015-05-21	Generic pharmaceutical business & certain assets	Strides Shasun Ltd	Aspen Pharmacare Holdings Ltd	299.71	Cash		Completed
2015	2014-08-20	Gansu Chengji Bio Pharmaceutical Co Ltd	Hybio Pharmaceutical Co Ltd		214.98	Cash and Stock		Completed
2015	2015-02-24	Neutec Toplam Kalite Yonetimi Sanayi Ticaret AS	Takeda Pharmaceutical Co Ltd	Neutec Ilac Sanayi Ve Ticaret AS	121.26	Cash		Completed
2015	2015-01-27	Polytech-Domilens GmbH	Stirling Square Capital Partners LLP		107.7	Cash		Completed
2015	2015-07-17	Beijing Jiu He Pharmaceutical Ltd	CHINAGRANDPHARM	Ningbo CDH Jinxiu Investment Management Co Ltd	72.8	Cash		Completed
2015	2014-10-15	Litha Healthcare Group Ltd	Endo International PLC		41.59	Cash	11.57	Completed
2015	2015-07-07	Guangzhou Pharmaceutical Research Institute Co Ltd	BAIYUNSHAN PH	Guangzhou Pharmaceutical Holdings Ltd	25.79	Cash		Completed
2015	2015-03-17	4 targeted oncology development programs worldwide	Ignyta Inc	Teva Pharmaceutical Industries Ltd	11.44	Stock		Completed
2015	2015-08-12	Semi-occlusive wound dressing	Madison Ventures Inc	Ocure Ltd	0.28	Cash		Completed
2015	2015-04-10	Aurobindo Pharma Australia Pty Ltd	Allergan plc	Aurobindo Pharma Ltd	N/A	Undisclosed		Completed
2015	2015-02-10	RAK Pharmaceuticals Pvt Ltd	Gulf Pharmaceutical Industries PSC	RAK Ceramics Bangladesh Ltd	N/A	Undisclosed		Completed
2015	2015-10-28	Acris Antibodies GmbH	OriGene Technologies Inc		N/A	Undisclosed		Completed
2015	2015-10-14	Primm Pharma SRL	Xbrane Bioscience AB		N/A	Undisclosed		Completed
2015	2015-09-16	Medicamen Biotech Ltd	Shivalik Rasayan Ltd		N/A	Cash		Completed
2015	2015-11-25	Groupe LCD SRL	Biogroup SELAFA		N/A	Undisclosed		Completed
2015	2015-11-09	International Pharmaceutical Generics Ltd	Emcure Pharmaceuticals USA Inc		N/A	Undisclosed		Completed
2014	2013-10-02	Acino International AG	[illegible]: Nordic Capital Fund VII LP),Avista Capital Holdings LP (Fund: Avista Capital Partners III LP)		577.95	Cash	12.39	Completed
2014	2014-01-16	Alvogen Korea Ltd/Old	Lotus Pharmaceutical Co Ltd	Alvogen Asia Pacific Holdings Ltd	157.24	Cash		Completed
2014	2014-04-01	Silom Medical International Co Ltd	Allergan plc		100	Cash		Completed
2014	2014-12-08	Oriola-KD's Russian businesses	Apteki 366 OOO	Oriola-KD OYJ	68.73	Cash		Completed
2014	2014-02-28	Australia OTC products	Perrigo Co PLC	Aspen Global Inc	51	Cash		Completed
2014	2014-07-21	India branded generics business	Strides Shasun Ltd	Bafna Pharmaceuticals Ltd	7.98	Cash		Completed
2014	2014-06-03	KPX Bio Tech Co Ltd	Sungwun Pharmacopia Co Ltd	Private Investor,KPX Holdings Corp	5.98	Cash		Completed
2014	2014-05-15	OrganiGram Inc	OrganiGram Holdings Inc		3.28	Stock		Completed
2014	2014-10-06	Vida Laboratories Ltd	Private Investor	Silk Road Energy Services Group Ltd	2.06	Cash		Completed
2014	2014-07-18	Quantum Healthcare Thailand Co Ltd,Oncology Imaging Systems Korea Co Ltd	Quantum Energy Ltd		N/A	Undisclosed		Completed
2014	2014-02-03	Nanomi BV	Lupin Ltd		N/A	Undisclosed		Completed
2014	2014-12-03	WindStar Medical GmbH	HQ Capital Private Equity LLC		N/A	Cash		Completed
2014	2014-05-01	Otaniguchi Pharmacy Business Units	Weeds Co Ltd	Otaniguchi Pharmacy KK	N/A	Undisclosed		Completed
2014	2014-11-20	Huvepharma EOOD	Advance Properties Ood	TRG Management LP	N/A	Cash		Completed
2014	2014-06-19	Sunsho Pharmaceutical Co Ltd	Carlyle Group LP/The		N/A	Undisclosed		Completed
2013	2013-08-13	Bever Pharmaceutical Pte Ltd	Pharmstandard PJSC		590	Cash and Stock		Completed

2013	2013-03-11	Simcere Pharmaceutical Group	Group,Right Lane Ltd,King View Development International Ltd,New Good Management Ltd,Assure Ahead Investm		114.56	Cash	11.98	Completed
2013	2011-08-30	Sanitas AB	Valeant Pharmaceuticals International Inc		57.76	Cash	12.94	Completed
2013	2013-06-04	Novavax AB	Novavax Inc		26.48	Stock		Completed
2013	2013-04-30	Pitney Pharmaceuticals Pty Ltd	PharmAust Ltd		4.55	Stock		Completed
2013	2013-08-29	Beijing Lingrui Sanity Material Co Ltd	Henan Lingrui Group Co Ltd	Henan Lingrui Pharmaceutical Co	3.1	Cash		Completed
2013	2013-02-13	OTC Pharmaceutical assets	Valeant Pharmaceuticals International Inc	Lek-Am Sp zoo	N/A	Cash		Completed
2013	2013-01-15	Labormed-Pharma SA	Alvogen Inc	Advent International Corp	N/A	Undisclosed		Completed
2013	2013-07-24	Opalia Pharma	Recordati SpA		N/A	Cash		Completed
2013	2013-10-23	CBD Life Sciences Inc	GelStat Corp		N/A	Undisclosed		Completed
2013	2013-11-01	CALADRYL	Piramal Enterprises Ltd	Valeant Pharmaceuticals International Inc	N/A	Cash		Completed
2013	2013-12-23	Sogeval SA	Ceva Sante Animale SA	Sofiproteol	N/A	Undisclosed		Completed
2013	2013-11-04	Opalia Pharma	Recordati SpA	Abraaj Group Ltd/The	N/A	Cash		Completed
2013	2013-12-16	ZellBios SA	DPE Deutsche Private Equity GmbH	Ergon Capital Partners SA	N/A	Cash		Completed
2012	2012-04-25	Actavis Group HF	Allergan plc	Novator EHF	5610.42	Cash		Completed
2012	2012-02-21	Pharmaplan Pty Ltd	Litha Healthcare Group Ltd	Paladin Labs Inc	78.85	Cash and Stock		Completed
2012	2011-05-23	Par Formulations Pvt Ltd	Par Pharmaceutical Cos Inc		37.6	Cash		Completed
2012	2012-02-24	Suzhou First Pharmaceutical Co Ltd	China NT Pharma Group Co Ltd	Suzhou Pharmaceutical Group Co Ltd	9.53	Cash		Completed
2012	2012-07-10	Anhui Wei Na Shengmingkexue Jishu Kai Fa	Daito Pharmaceutical Co Ltd		7	Cash		Completed
2012	2012-04-18	Certain Assets	Valeant Pharmaceuticals International Inc	Atlantis SA de CV	5.4	Cash		Completed
2012	2012-10-02	Macleods Pharmaceuticals Ltd	Neogen Corp		N/A	Undisclosed		Completed
2012	2012-05-29	Exclusive Rights to Intellectual Property	Raptor Pharmaceutical Corp	McGill University	N/A	Undisclosed		Completed
2012	2012-07-04	YPA KK	Kamei Corp		N/A	Undisclosed		Completed
2012	2012-02-21	Pharmaplan Pty Ltd	Paladin Labs Inc		N/A	Undisclosed		Completed
2012	2012-07-03	WISAP Medical Technology GmbH	Blue Cap AG		N/A	Cash		Completed
2011	2011-05-19	Nycomed A/S	Takeda Pharmaceutical Co Ltd	Nordic Capital Fund V LP),Avista Capital Holdings L	13732.8	Cash		Completed
2011	2011-05-24	Sanitas AB	Valeant Pharmaceuticals International Inc	l International (Fund: TRG Growth Partnership LP),Ci	463.05	Cash	11.86	Completed
2011	2011-09-26	Teva-Kowa Pharma Co	Teva Pharmaceutical Industries Ltd	Kowa Co Ltd/Aichi	150	Cash		Completed
2011	2011-08-01	C&O Pharmaceutical Technology Holdings Ltd	Shionogi & Co Ltd		128.58	Cash	8.63	Completed
2011	2011-01-25	Tenth of Ramadan Pharmaceuticals and Diagnostic Re	tments,Sphinx Private Equity Management (Fund: Sphinx Turnaround Fund),Compass Capital		40	Cash		Completed
2011	2011-06-01	Flower Pharmacy Tamashiro	Medical Ikkou Co Ltd		N/A	Cash		Completed
2011	2011-06-17	China Resources Purenhong Beijing Pharmaceutical C	China Resources Pharmaceutical Commercial Group Co		N/A	Undisclosed		Completed
2011	2011-06-06	NextEra As	enture AS (Fund: Birk Venture/Fund),Olsen Capital & Consulting AS		N/A	Cash		Completed
2011	2011-07-08	Certain Assets	CH Boehringer Sohn AG & Co KG	Svanova Biotech AB	N/A	Cash		Completed
2011	2011-08-02	Certain Assets	SOHM Inc	Novatrend Medicament Pvt Ltd	N/A	Cash		Completed
2011	2011-09-01	Clover YK	Medical Ikkou Co Ltd		N/A	Undisclosed		Completed
2011	2011-01-19	Biolek	Pharmstandard PJSC		N/A	Cash		Completed
2011	2011-03-25	Exclusive license	XTL Biopharmaceuticals Ltd	MinoGuard	N/A	Cash		Completed
2011	2011-11-15	ADDvance Brand	Adcock Ingram Holdings Ltd	Peppina Sales	N/A	Cash		Completed
2011	2011-01-19	Biolek	Farmstandart OAO		N/A	Undisclosed		Completed
2011	2011-10-17	Square Cephalosporins Ltd	Square Pharmaceuticals Ltd		N/A	Undisclosed		Completed
2010	2009-11-05	Swedish Orphan International AB	Swedish Orphan Biovitrum AB		500.25	Cash		Completed
2010	2010-10-28	ANB Laboratories Co Ltd	Bangkok Dusit Medical Services PCL		24.41	Cash		Completed
2010	2009-11-18	Israel Cosmetics Business	Sano-Brunos Enterprises Ltd	Emilia Development Ltd	22.56	Cash		Completed
2010	2010-05-11	Nihon Pharmaceutical Industry Co Ltd	Nippon Chemiphar Co Ltd		12.4	Stock		Completed
2010	2008-06-30	Taro Pharmaceutical Industries Ltd	Sun Pharmaceutical Industries Ltd		0.2	Cash		Completed
2010	2010-05-07	Manufacturing Facilities at Kurkumbh	Cipla Ltd/India		N/A	Cash		Completed
2010	2010-06-18	Medisa Shinyaku Inc	Sawai Pharmaceutical Co Ltd	Sumitomo Corp	N/A	Undisclosed		Completed
2010	2010-08-03	Pharmaceutical Products	AA Pharma	Apotex Inc	N/A	Cash		Completed
2010	2010-09-02	Vindexpharm ZAO	Pharmstandard PJSC		N/A	Cash		Completed
2010	2010-09-30	Metoprolol Succinate Product	Intas Pharmaceuticals Ltd	Orbus Pharma Inc	N/A	Cash		Completed
2010	2010-09-30	Daichi Kasei Co Ltd	Towa Pharmaceutical Co Ltd	Private Investor,KNC Laboratories Co Ltd	N/A	Undisclosed		Completed
2010	2010-12-23	Confab Laboratories Inc	RoundTable Healthcare Partners		N/A	Cash		Completed
2010	2010-12-07	Biotec Services International Ltd	nt Group,Finance Wales Investments Ltd (Fund: Wales JEREMIE Fund)		N/A	Cash		Completed
2009	2008-06-18	Zentiva NV	Sanofi		2943.38	Cash	14.39	Completed
2009	2009-01-26	Farma APS	nent Group,Magnum Industrial Partners SL (Fund: Magnum Capital LP)		236.63	Cash		Completed
2009	2008-10-16	al Laboratories Teva Ltd Israel,Abic Veterinary Products Ltd Israel,Assia Pharmaceutic	Phibro Animal Health Corp	Teva Pharmaceutical Industries Ltd	47	Cash		Completed
2009	2009-08-28	Jiangxi Hangtian Tailishi Pharmaceutical Co Ltd	Renhe Pharmacy Co Ltd	Investor,Nanchang Hangtian Investment Developmen	2.64	Cash		Completed
2009	2009-03-31	Beijing Univision Pharmaceutical Co Ltd	Center Laboratories Inc		0.1	Cash		Completed
2009	2009-04-02	Laboratorios Kendrick SA	Sanofi		N/A	Undisclosed		Completed
2009	2009-03-19	asi Saglik Urunleri Sanayi ve Ticaret AS,EOS Eczacibasi Ozgun Kimyasal Urunler Sana	Zentiva NV	EIS Eczacibasi Ilac ve Sinai ve Finansal Yatirimla	N/A	Undisclosed		Completed
2009	2009-08-03	Portfolio of 20 branded generic products	Nycomed SCA SICAR	Sanofi	N/A	Cash		Completed
2009	2009-07-03	Ostelin brands	Sanofi	Reckitt Benckiser Group PLC	N/A	Cash		Completed
2009	2008-12-09	Generis-Farmaceutica SA	Magnum Industrial Partners SL		N/A	Cash		Completed
2009	2009-07-24	Product Portfolio & Ozone Brand & Intellectual Pro	Advent International Corp	Ozone Laboratories Group	N/A	Cash		Completed
2008	2008-07-21	Symbion Consumer	Sanofi	Primary Health Care Ltd	545.83	Cash		Completed
2008	2007-12-24	Orphan Holdings Pty Ltd	Sigma Pharmaceuticals Ltd		113.26	Cash		Completed

2008	2008-11-25	Generic Pharmaceutical Products	Allergan plc	Teva Pharmaceutical Industries Ltd	36	Cash		Completed
2008	2008-06-04	Oncology Projects	4SC AG	Nordic Capital Svenska AB	21.64	Cash		Completed
2008	2008-04-22	Wuhu Zhong Ren Pharmaceutical Co Ltd	Simcere Pharmaceutical Group		9.27	Cash		Completed
2008	2008-08-25	Immunocorp Animal Health AS	Zilor Group	Biotec Pharmacon ASA	6.99	Cash		Completed
2008	2008-04-16	Nanjing Xinaokang Pharmaceutical Ltd	C&O Pharmaceutical Technology Holdings Ltd		5.72	Cash		Completed
2008	2008-10-20	Lianyungang Kangyuan Pharmacetical Commercial Co L	Jiangsu Jinglue Enterprise Development Co Ltd	Jiangsu Kanion Pharmaceutical Co Ltd	5.29	Cash		Completed
2008	2007-10-03	Zenotech Laboratories Ltd	Ranbaxy Laboratories Ltd		2.56	Cash	123.58	Completed
2008	2007-12-17	Sun Pharmaceutical Sdn Bhd	Sunway Holdings Sdn Bhd		0.75	Cash		Completed
2008	2008-07-10	Sichuan Changao Medicine Co Ltd	C&O Pharmaceutical Technology Holdings Ltd		0.01	Cash		Completed
2008	2008-03-12	ai Huayuan Pharmaceutical Sale Co Ltd,Shanghai Huayuan Pharmaceutical Technolog	Private Investor	Zhuhai Boyuan Investment Co Ltd	N/A	Cash		Completed
2008	2007-11-15	Eduard Vogt AG	Tentan AG	Galenica AG	N/A	Undisclosed		Completed
2008	2007-12-07	Laboratorio Sanderson SA	Fresenius SE & Co KGaA		N/A	Undisclosed		Completed
2008	2008-04-03	Dr Reddy's Srl	Dr Reddy's Laboratories Ltd		N/A	Undisclosed		Completed
2008	2008-04-17	Altisana UAB	Unnamed Buyer	Sanitas AB	N/A	Undisclosed		Completed
2008	2008-04-24	Tamda SA	Fagron		N/A	Undisclosed		Completed
2008	2008-05-28	Aquaworx AG	aligna AG		N/A	Cash and Stock		Completed
2008	2008-04-30	Plasmaverarbeitungsgesellschaft	Octapharma AG		N/A	Undisclosed		Completed
2008	2007-03-01	Orphan Pharma International Ltd	EUSA Pharma Ltd		N/A	Undisclosed		Completed
2008	2008-10-02	Budesonide Formulation	OPKO Health Inc	Teva Pharmaceutical Industries Ltd	N/A	Cash		Completed
2008	2008-12-23	von der Linde GmbH & Co Immobilienverwaltung KG	Sanacorp Pharmahandel GmbH		N/A	Undisclosed		Completed
2007	2007-05-12	Merck Generics	Mylan NV	Merck KGaA	6620.88	Cash		Completed
2007	2007-05-10	Actavis Group HF	Novator EHF		4542.59	Cash	16.2	Completed
2007	2006-09-21	Hospira Australia Pty Ltd	Hospira Inc		1912.3	Cash	15.89	Completed
2007	2007-03-05	asi Saglik Urunleri Sanayi ve Ticaret AS,EOS Eczacibasi Ozgun Kimyasal Urunler Sana	Zentiva NV	EIS Eczacibasi Ilac ve Sinai ve Finansal Yatirimla	602.14	Cash		Completed
2007	2006-11-08	Plant in Spain	Lonza Group AG	Genentech Inc	191.51	Cash		Completed
2007	2007-11-09	TAD Pharma GmbH	Krka dd Novo mesto	PHW Gruppe Lohman & Co AG	142.28	Cash		Completed
2007	2007-08-31	Forum Bioscience Holdings Ltd	STADA Arzneimittel AG	Ajinomoto Co Inc	76.03	Cash		Completed
2007	2007-09-07	Generics business	Novator EHF	aligna AG	75.08	Cash		Completed
2007	2007-03-05	Cerbo Group AB	Nolato AB	Vision Capital Group Ltd	61.44	Cash		Completed
2007	2007-09-05	ORCA Pharm GmbH	PLIVA Farmaceutika DD		28.71	Cash		Completed
2007	2007-09-05	ORCA Pharm GmbH	Barr Pharmaceuticals Inc		28.57	Cash		Completed
2007	2007-06-11	Grandix Pharmaceuticals Ltd	Strides Shasun Ltd		24.5	Cash		Completed
2007	2008-01-02	Dr Fisher Farma BV	Mosadex CV	SnowWorld NV	19.74	Cash		Completed
2007	2007-08-28	Biomeda Group	Elder Pharmaceuticals Ltd		6.82	Cash		Completed
2007	2007-06-07	Yangzhou Uni-bio Pharmaceutical Co Ltd	Jinyu Bio-Technology Co Ltd		2.94	Cash		Completed
2007	2007-05-03	Patents	Genovis AB	Biolin Scientific AB	0.3	Cash		Completed
2007	2007-05-02	Pharmatec GmbH	Robert Bosch GmbH	Fresenius SE & Co KGaA	N/A	Undisclosed		Completed
2007	2007-07-09	Wildlife DNA Services Ltd,Food DNA Services Ltd	Hologic Ltd		N/A	Undisclosed		Completed
2007	2007-12-11	Ribbon	Fresenius SE & Co KGaA		N/A	Undisclosed		Completed
2007	2007-04-19	Shenzhou Tongde Pharmaceutical Co Ltd	Zoetis Products LLC		N/A	Undisclosed		Completed
2007	2007-05-09	Zalemark Holding Co Inc	Charis Industries Co		N/A	Undisclosed		Completed
2006	2006-03-28	Sindan	Actavis Group HF		161.23	Cash		Completed
2006	2006-08-14	Adeerall tablets	Barr Pharmaceuticals Inc	Shire PLC	63	Cash		Completed
2006	2006-04-20	Nagoya Plaza Building	Ichigo Real Estate Investment Corp/Old	Taiyo Yakuhin Co Ltd	49.4	Cash		Completed
2006	2006-02-01	Provalis Healthcare Ltd	Kogen Ltd	Provalis PLC	18.66	Cash		Completed
2006	2006-02-27	Shenzhen Liancheng Medicine Co Ltd	C&O Pharmaceutical Technology Holdings Ltd		5.6	Cash		Completed
2006	2006-08-07	PB Diagnostics Ltd	Bio-Rad Laboratories Inc	Provalis PLC	3.05	Cash		Completed
2006	2006-06-23	Patent rights	Sanofi Pasteur Ltd	Provalis PLC	1.25	Cash		Completed
2006	2006-01-11	Nanjing Changao Pharmaceutical Science & Technolog	C&O Pharmaceutical Technology Holdings Ltd		0.09	Cash		Completed
2006	2005-12-15	Cyclacel Ltd	Cyclacel Pharmaceuticals Inc	Cyclacel Group PLC	N/A	Undisclosed		Completed
2006	2006-12-20	Manufacturing plant	Actavis Group HF	Grandix Pharmaceuticals Ltd	N/A	Cash		Completed
2006	2006-12-25	Shanghai Weike Bio Pharmaceutical Co Ltd	Sanjiu Enterprise Group	Zhenxing Biopharmaceutical and Chemical Co Ltd	N/A	Cash		Completed
2006	2008-01-23	Masterlek	Pharmstandard PJSC		N/A	Undisclosed		Completed
2005	2005-02-21	Hexal AG	Novartis AG		5685.89	Cash		Completed
2005	2005-08-25	Docpharma BVBA	Mylan Laboratories Ltd		263.18	Cash		Completed
2005	2005-04-22	Clifford Hallam Pharmaceuticals Pty Ltd	Joint Venture	Spotless Group Ltd	52.74	Cash		Completed
2005	2004-03-24	Oriental Wave Holding Ltd	Dragon Pharmaceutical Inc		41.75	Undisclosed		Completed
2005	2005-03-07	CIMEX Pharma AG	Acino International AG		26.98	Cash	13.37	Completed
2005	2005-04-04	Ipsat Therapies Oy	Co,Bio Fund Management Oy,Finnish Industry Investment Ltd,Sitra the Finnish Innovation Fund		8.99	Cash		Completed
2005	2005-06-15	Beximco Infusions Ltd	Beximco Pharmaceuticals Ltd		N/A	Stock		Completed
2005	2005-09-30	Keri Pharma Generics	Actavis Group HF	Keri Pharma	N/A	Undisclosed		Completed
2005	2005-01-25	Laboratorios Kendrick SA	Darby Overseas Investments Ltd		N/A	Undisclosed		Completed
2005	2005-09-01	Biotec Pharmacon ASA	Management Group	NorgesInvestor AS	N/A	Undisclosed		Completed
2005	2005-03-10	Nycomed Holding ApS	Nordic Capital Svenska AB	Partners III LP),AlpInvest Partners BV,Blackstone G	N/A	Cash		Completed
2004	2004-06-07	Sabex Holdings Ltd	Novartis AG		565.97	Cash		Completed
2004	2004-12-07	CIMEX Pharma AG	Acino International AG		112.79	Cash and Stock	13.07	Completed

2004	2004-08-17	Dorom Srl	Teva Pharmaceutical Industries Ltd	Pfizer Inc	85.18	Cash		Completed
2004	2004-07-13	Biovena Pharma Sp zoo	Actavis Group HF		8.62	Cash		Completed
2004	2004-07-22	Aquatic animal health operations	Investor Group	Zoetis Products LLC	3.9	Undisclosed		Completed
2004	2004-07-30	Diaclone Research	Hologic Ltd	Orphan Pharma International Ltd	2.85	Undisclosed		Completed
2004	2004-12-23	Riasima Abadi Farma PT	Trimarga Rekatama PT	Indofarma Persero Tbk PT	0.62	Cash		Completed
2004	2004-01-05	Unnamed Target/CN	Bio-One Corp		N/A	Cash and Stock		Completed
2004	2003-12-13	RPG Aventis SA	Ranbaxy Laboratories Ltd	Sanofi-Aventis SA	N/A	Undisclosed		Completed
2004	2004-04-29	Katwijk Farma BV	Apotex Inc	UBS AG	N/A	Undisclosed		Completed
2004	2004-07-19	Linotar & Exorex	Clinuvel Pharmaceuticals Ltd	TransDermal Pharmaceuticals Australia Pty Ltd	N/A	Undisclosed		Completed
2003	2003-04-02	CLL Pharma SA	Neuro Bioscience Inc		43.81	Stock		Completed
2003	2003-04-30	Sanitas AB	Kremi UAB		7.35	Cash		Completed
2003	2003-04-04	Serviphar	Docpharma BVBA		4.13	Cash and Stock		Completed
2003	2003-07-24	Apothecon BV	Docpharma BVBA		1.99	Cash and Stock		Completed
2003	2003-04-11	Natural White Inc	Natural White Holdings Ltd		N/A	Undisclosed		Completed
2003	2003-04-29	Diaclone	Orphan Pharma International Ltd	Biotest AG	N/A	Undisclosed		Completed
2003	2003-02-18	Roscrea Pharmaceutical facility	Taro Pharmaceutical Industries Ltd		N/A	Undisclosed		Completed
2002	2002-10-30	Nycomed Holding ApS	king Partners III LP),AlpInvest Partners BV,Blackstone Grou	Nordic Capital Svenska AB	1125.82	Cash		Completed
2002	2002-03-12	Dr Reddy's Laboratories EU Ltd	Dr Reddy's Laboratories Ltd		12.81	Undisclosed		Completed
2002	2002-02-25	2K Pharmaceuticals AS	PLIVA Farmaceutika DD		3.57	Cash		Completed
2002	2001-06-20	Mohan Medicine Research Institute	Merck KGaA	Kyowa Hakko Kirin Co Ltd	N/A	Undisclosed		Completed
2002	2002-04-02	Laboratoires Merck Sharp & Dohme-Chibret SNC	IVAX Corp	Merck & Co Inc	N/A	Undisclosed		Completed
2002	2002-09-03	Eduard Vogt AG	Galenica AG		N/A	Undisclosed		Completed
2001	2001-07-11	Actavis Elizabeth LLC	Zoetis Products LLC	Idameneo No.789 Ltd	660	Cash		Completed
2001	2001-08-24	Nycomed Holding ApS	Nordic Capital Svenska AB	GE Healthcare Ltd	177.48	Cash		Completed
2001	2001-09-12	Aktuapharma SA	Docpharma BVBA		4.39	Cash and Stock		Completed
2001	2000-12-21	Labinca SA	Novartis AG		N/A	Undisclosed		Completed
2001	2001-04-02	Lagap Pharmaceuticals Ltd	Novartis AG	Tiger Brands Ltd	N/A	Undisclosed		Completed
2001	2001-06-27	2 Latvia drug wholesalers	Tamro OYJ		N/A	Undisclosed		Completed
2000	2000-05-30	Technilab Pharma Inc	Merckle GmbH		51.88	Cash	8.42	Completed
2000	2000-10-13	Laporte Organics Francis SpA	Dipharma SpA	Laporte PLC	14.58	Undisclosed		Completed
2000	2000-09-08	Fagron Group	Omega Pharma NV		N/A	Undisclosed		Completed
1999	1999-05-05	Nycomed Holding ApS	Nordic Capital Svenska AB	GE Healthcare Ltd	450.07	Cash		Completed
1998	no deals							
1997	no deals							
1996	no deals							
1995	no deals							

YEAR	ANNOUNCED VALUE (MIL)	ANNOUNCED VALUE (BIL)	# OF DEALS
2016	1064.09	1.06	25
2015	6898.25	6.90	18
2014	974.22	0.97	15
2013	796.45	0.80	14
2012	5748.80	5.75	11
2011	14514.43	14.51	16
2010	559.82	0.56	13
2009	3229.75	3.23	11
2008	747.32	0.75	22
2007	14335.83	14.34	21
2006	302.28	0.30	12
2005	6079.53	6.08	11
2004	779.93	0.78	11
2003	57.28	0.06	7
2002	1142.20	1.14	6
2001	841.87	0.84	6
2000	66.46	0.07	3
1999	450.07	0.45	1
1998	0.00	0.00	0
1997	0.00	0.00	0
1996	0.00	0.00	0
1995	0.00	0.00	0
**Total**	**58588.58**	**58.59**	**223**
