# Supplementary material for: Merger mania: mergers and acquisitions in the generic drug sector from 1995 to 2016
Source: Global Health. 2017 Aug 22;13:62. doi: 10.1186/s12992-017-0285-x (PMC5567637; doi:10.1186/s12992-017-0285-x)
Supplement: Supplementary file 1 — Global, United States, and Global excluding the United States deal specific data. (PDF 313 KB) [file 12992_2017_285_MOESM1_ESM.zip › 12992_2017_285_MOESM1_ESM/Deal specific data USA.pdf]

Completed-Year	Announce Date	Target Name	Acquirer Name	Seller Name	Announced Total Value (mil.)	Payment Type	TV/EBITDA	Deal Status
1995	no deals							
1996	1996-01-29	Biocraft Laboratories Inc	Teva Pharmaceutical Industries Ltd		356.1	Stock		Completed
2016	2015-07-27	Generic drug business	Teva Pharmaceutical Industries Ltd	Allergan plc	39564	Cash and Stock		Completed
2016	2016-05-13	Generics Business/Illinois	Mylan NV	Renaissance Acquisition Holdings LLC	950	Cash		Completed
2016	2015-07-23	Gavis Pharmaceuticals LLC,Novel Laboratories Inc	Lupin Ltd		880	Undisclosed		Completed
2016	2016-03-29	Epic Pharma LLC,Epic RE Holdco LLC	Humanwell Healthcare Group Co Ltd		550	Cash		Completed
2016	2015-09-04	InvaGen Pharmaceuticals Inc,Exelan Pharmaceuticals Inc	Cipla Ltd/India		550	Cash		Completed
2016	2016-06-11	Portfolio of 8 abbreviated new drug applications	Dr Reddy's Laboratories Ltd	eva Pharmaceutical Industries Ltd,Allergan p	350	Cash		Completed
2016	2016-01-07	Avioq Inc	Shandong Oriental Ocean Sci-Tech Co Ltd		68.22	Cash		Completed
2016	2016-05-03	KYNAMRO develop & commercialize rights	Kastle Therapeutics LLC	Ionis Pharmaceuticals Inc	25	Cash and Stock		Completed
2016	2016-04-27	Biozone Laboratories Inc	Flavor Producers Inc	MusclePharm Corp	8.3	Cash		Completed
2016	2016-02-24	ADASUVE US Commerical Rights	Alexza Pharmaceuticals Inc	Teva Pharmaceutical Industries Ltd	0.67	Stock		Completed
2016	2016-02-11	Beyond Human LLC	Innovus Pharmaceuticals Inc		0.63	Cash		Completed
2016	2016-06-24	Smith Brothers Co/The	GR Lane Holdings Ltd		N/A	Undisclosed		Completed
2016	2016-06-20	US VMS Business	International Vitamin Corp	Perrigo Co PLC	N/A	Cash		Completed
2016	2016-07-26	Ibudtender LLC	Cannabis Sativa Inc		N/A	Undisclosed		Completed
2016	2016-10-06	Convey Health Solutions Holdings LLC	New Mountain Capital LLC	ComVest Group Inc	N/A	Cash		Completed
2016	2015-10-21	7 brands/Johnson & Johnson	Strides Shasun Ltd	Johnson & Johnson	N/A	Cash		Completed
2016	2016-12-29	ept pregnancy test kit brand	NFI Products Inc	Prestige Brands Holdings Inc	N/A	Cash		Completed
2015	2015-02-05	Hospira Inc	Pfizer Inc		16807.25	Cash	23.2	Completed
2015	2015-05-18	Par Pharmaceutical Holdings Inc	Endo International PLC	artners VI LP),TPG Capital Management LP	8090.04	Cash and Stock	32.49	Completed
2015	2014-10-09	Lineage Therapeutics Inc,Tower Holdings Inc	Impax Laboratories Inc	able Healthcare Partners (Fund: Roundtable	691.3	Cash		Completed
2015	2015-02-05	Branded respiratory business in the US and Canada	AstraZeneca PLC	Allergan plc	600	Cash		Completed
2015	2015-08-24	Talazoparib Drug Rights	Medivation Inc	BioMarin Pharmaceutical Inc	410	Cash		Completed
2015	2015-05-12	Pantheon's Mexican operations	Perrigo Co PLC	Patheon Inc	34	Cash		Completed
2015	2015-07-13	22 generic drug products	ANI Pharmaceuticals Inc	Teva Pharmaceutical Industries Ltd	25	Cash		Completed
2015	2015-04-09	CynoGen Inc	Rosetta Genomics Ltd	Prelude Corp	3.51	Cash and Stock		Completed
2015	2015-03-30	Domestic Generic Pharmaceuticals Business	Amneal Pharmaceuticals LLC	Allergan plc	N/A	Undisclosed		Completed
2015	2015-06-30	Time-Cap Laboratories Inc	Marksans Pharma Ltd		N/A	Undisclosed		Completed
2015	2015-07-20	OmniLytics Inc	Phagelux Inc		N/A	Undisclosed		Completed
2015	2015-02-12	PDX business unit	Crown Bioscience Inc	Molecular Response LLC	N/A	Cash		Completed
2015	2015-12-03	MSD's Portfolio of Glaucoma Products	Mundipharma Ophthalmology Products Ltd	Merck & Co Inc	N/A	Cash		Completed
2015	2015-12-14	Bryan Ohio unit/Sun Pharmaceutical Industries Ltd	Mylan NV	Sun Pharmaceutical Industries Ltd	N/A	Cash		Completed
2015	2015-06-22	Alvogen Inc	k Holdings Pte Ltd,CVC Capital Partners Ltd,Vatera Healthcare Partr	Pamplona Capital Management LLP	N/A	Cash		Completed
2015	2015-03-02	Sellersville facility	G&W Laboratories Inc	Teva Pharmaceutical Industries Ltd	N/A	Cash		Completed
2014	2014-06-24	DAVA Pharmaceuticals Inc	Endo International PLC		575	Cash		Completed
2014	2013-08-28	Boca Pharmacal Inc	Endo International PLC		225	Cash		Completed
2014	2014-03-27	PACK Pharmaceuticals LLC	Aceto Corp		85	Cash and Stock		Completed
2014	2014-04-02	Alvogen Inc	Pamplona Capital Management LLP		N/A	Cash		Completed
2014	2014-04-17	Four branded ophthalmic and topical products	Allergan plc	Akorn Inc	N/A	Cash		Completed
2014	2014-04-16	Antioxidant ibuprofen and propofol businesses	SI Group Inc	Albemarle Corp	N/A	Cash		Completed
2014	2014-01-13	CGRP antibody	Eli Lilly & Co	Arteaus Therapeutics LLC	N/A	Cash		Completed
2013	2013-05-01	Metronidazole 1.3% Vaginal Gel	Allergan plc	Valeant Pharmaceuticals International Inc	55	Cash		Completed
2013	2013-12-26	31 generic drug products	ANI Pharmaceuticals Inc	Teva Pharmaceutical Industries Ltd	12.5	Cash		Completed
2013	2013-07-02	Libertas Pharma Inc	Mayne Pharma Group Ltd		N/A	Undisclosed		Completed
2013	2013-09-30	4 generic medical products	Amneal Pharmaceuticals LLC	Allergan plc	N/A	Cash		Completed
2013	2013-11-07	Naprelan rights	Alvogen Inc	Shionogi & Co Ltd	N/A	Cash		Completed
2013	2013-12-16	ubilant Cadista Pharmaceuticals Inc,Vertical Pharmaceuticals I	Avista Capital Holdings LP		N/A	Undisclosed		Completed
2012	2012-07-16	Par Pharmaceutical Cos Inc	Fund: TPG Partners VI LP),TPG Capital Management LP (Fund: TPG Biotechnology Partners IV LP)		1934.2	Cash	9.86	Completed
2012	2012-10-29	Banner Pharmacaps Inc	Patheon Inc	Vion NV	255	Cash		Completed
2012	2012-12-28	Cobrek Pharmaceuticals Inc	Perrigo Co PLC		45	Cash		Completed
2012	2012-01-17	Oncogenerix Inc	Midatech Pharma US Inc		1.45	Stock		Completed
2011	2011-01-20	Substantially all assets	Perrigo Co PLC	Paddock Laboratories Inc	540	Cash		Completed
2011	2011-08-24	Anchen Pharmaceuticals Inc	Par Pharmaceutical Cos Inc		410	Cash		Completed
2011	2011-07-07	one Laboratories Inc,Equachem LLC,Equalan Pharmaceuticals	Cocrystal Pharma Inc		104.98	Stock		Completed
2011	2010-12-06	Caraco Pharmaceutical Laboratories Ltd	Sun Pharmaceutical Industries Ltd		46.8	Cash		Completed
2011	2011-05-15	Land & Building	Unnamed Buyer	Allergan plc	4.7	Cash		Completed
2011	2011-06-15	Home Wellness Inc	Riverside Co/The		N/A	Cash		Completed
2011	2011-09-01	Detach N' Go	Apex Medical Corp/United States	Inventures Products LLC	N/A	Cash		Completed
2010	2010-09-28	Qualitest and Vintage Pharmaceuticals	Endo International PLC	Apax Partners LLP	1200	Cash		Completed
2010	2010-03-04	CRINONE Progesterone Gel Product Line	Allergan plc	Juniper Pharmaceuticals Inc	47	Cash		Completed

2010	2010-08-06	Apredica LLC	Cyprotex PLC		5.87	Cash and Stock		Completed
2010	2010-05-05	Certain Assets	Perrigo Co PLC		N/A	Cash		Completed
2010	2010-05-26	Intellectual Ppty & Assets	Perrigo Co PLC	Novel Laboratories Inc	N/A	Cash		Completed
2009	2009-12-30	Immunocorp Consumer Health AS	Sana Pharma AS	Biotec Pharmacon ASA	5.43	Cash		Completed
2009	2009-01-28	RxElite Holdings Inc	Piramal Enterprises Ltd	RXElite Inc	4.2	Cash		Completed
2009	2009-02-25	INVO BioScience Inc/Old	INVO BioScience Inc		N/A	Stock		Completed
2009	2009-04-01	Certain Assets	Par Pharmaceutical Cos Inc	Marina Biotech Inc	N/A	Cash		Completed
2009	2009-11-30	Breckenridge Pharmaceutical Inc	Pensa Pharma SA		N/A	Undisclosed		Completed
2009	2009-02-18	Agennix Inc	GPC Biotech AG		N/A	Stock		Completed
2008	2008-07-18	Barr Pharmaceuticals Inc	Teva Pharmaceutical Industries Ltd		8833.22	Cash and Stock	11.96	Completed
2008	2008-09-11	Zoetis Products LLC	KP Pharmaceuticals LLC		1253.63	Cash		Completed
2008	2008-02-06	Active Pharmaceutical Ingredients business	3i Group PLC	Zoetis Products LLC	395	Cash		Completed
2008	2008-04-25	Interpharm Holdings Inc	Amneal Pharmaceuticals LLC		N/A	Undisclosed		Completed
2008	2008-01-03	Certain Assets	Amneal Pharmaceuticals LLC		N/A	Cash		Completed
2008	2008-12-30	Kadian	Novator EHF	KP Pharmaceuticals LLC	N/A	Cash		Completed
2007	2007-11-21	Reliant Pharmaceuticals Inc	GlaxoSmithKline PLC	Bay City Capital LLC	1650	Cash		Completed
2007	2006-11-30	Abrika Pharmaceuticals Inc	Actavis Group HF		112.67	Cash		Completed
2007	2007-01-09	Natural gas properties/MI	EV Energy Partners LP		71.6	Cash		Completed
2007	2007-09-24	Pediatric asthma development programs	AstraZeneca PLC	Verus Pharmaceuticals Inc	30	Cash		Completed
2007	2007-08-10	Intertech Bio	Breitling Energy Corp		N/A	Stock		Completed
2007	2007-09-17	Qualitest and Vintage Pharmaceuticals	Apax Partners LLP		N/A	Undisclosed		Completed
2007	2007-05-22	Rockford-Montgomery Labs Inc	IAHL Corp		N/A	Undisclosed		Completed
2006	2005-07-25	IVAX Corp	Teva Pharmaceutical Industries Ltd		7575.54	Cash or Stock	23.32	Completed
2006	2006-03-08	Parmed Pharmaceuticals LLC	Cardinal Health Inc	Zoetis Products LLC	40.1	Cash		Completed
2006	2005-04-04	Generic pharmaceutical co	Jubilant Life Sciences Ltd		8.25	Cash		Completed
2006	2006-07-11	Gateway Medical Systems Inc	Ba Research International		N/A	Cash and Stock		Completed
2006	2006-10-16	Blackhawk BioSystems Inc	Bio-Rad Laboratories Inc		N/A	Undisclosed		Completed
2005	2005-02-21	Eon Labs Inc	Novartis AG	Santo Holding Deutschland GmbH	1699.23	Cash	13.7	Completed
2005	2005-02-21	Eon Labs Inc	Novartis AG		889.39	Cash	15.03	Completed
2005	2005-10-17	Global generic business	Actavis Group HF	Zoetis Products LLC	810	Cash		Completed
2005	2005-05-20	Actavis Totowa LLC	Actavis Group HF		500	Cash		Completed
2005	2005-02-15	Phoenix Scientific Inc	IVAX Corp		271.85	Cash and Stock		Completed
2005	2005-11-04	Apokyn drug rights	Vernalis PLC	Mylan NV	23	Cash		Completed
2005	2005-06-21	Trace Genetics Inc	DNAPrint Genomics Inc		0.28	Stock		Completed
2005	2005-08-04	CorePharma LLC	RoundTable Healthcare Partners		N/A	Cash		Completed
2005	2005-12-22	8 Products	Par Pharmaceutical Cos Inc		N/A	Cash		Completed
2005	2005-12-23	Able Laboratories Inc	Sun Pharmaceutical Industries Ltd		N/A	Undisclosed		Completed
2005	2005-06-30	AXID Oral Solutions	Braintree Laboratories Inc	Reliant Pharmaceuticals Inc	N/A	Undisclosed		Completed
2004	2004-01-22	Neupogen/Neulasta royalties	Royalty Pharma AG	Memorial Sloan-Kettering Cancer Center	263	Cash		Completed
2004	2004-04-13	Kali Laboratories Inc	Par Pharmaceutical Cos Inc		135	Cash		Completed
2004	2004-03-09	Convey Health Solutions Holdings LLC	NationsHealth Inc		107.18	Stock		Completed
2004	2004-10-07	Vetco Inc	Prestige Brands International Inc		49.3	Undisclosed		Completed
2004	2003-09-11	Rights to Loestrin products/US & Canada	Barr Pharmaceuticals Inc	Chilcott UK Ltd	44.93	Cash		Completed
2004	2004-02-04	Caraco Pharmaceutical Laboratories Ltd	Sun Pharmaceutical Industries Ltd		37.57	Cash	17.58	Completed
2004	2004-12-28	Rights to Nordette	Barr Pharmaceuticals Inc	KP Pharmaceuticals LLC	12	Cash		Completed
2004	2004-01-29	Fenofibrate distribution rights	Reliant Pharmaceuticals Inc	CML HealthCare Inc	N/A	Undisclosed		Completed
2004	2004-06-30	OTC Portfolio	Lil' Drug Store Products Inc	Juniper Pharmaceuticals Inc	N/A	Undisclosed		Completed
2003	2002-11-26	Interpharm Inc	Interpharm Holdings Inc		N/A	Undisclosed		Completed
2003	2003-04-10	Accupac Inc	HIG Capital LLC		N/A	Undisclosed		Completed
2003	2003-10-29	Medicos Laboratories Inc	Inamco International Corp		N/A	Undisclosed		Completed
2003	2003-11-19	LiquiSource Inc	Able Laboratories Inc		N/A	Cash		Completed
2003	2003-03-13	Sea & Ski	Pathfinder Management Inc	Idameneo No.789 Ltd	N/A	Undisclosed		Completed
2002	2002-03-06	GeneThera Inc/Old	GeneThera Inc		N/A	Stock		Completed
2002	2002-08-13	Centex HomeTeam Services lawn care operations	Scotts Miracle-Gro Co/The	Centex LLC	N/A	Undisclosed		Completed
2001	2001-12-18	Reliant Pharmaceuticals Inc	y City Capital LLC (Fund: Bay City Capital Fund III LP),Versant Ventures Management LLC (Fund: Versant Ventu		150	Cash		Completed
2001	2000-10-31	Generic Distributors Inc	Unnamed Buyer	Able Laboratories Inc	N/A	Cash		Completed
2001	2001-03-29	Rights to certain ANDAs	Zoetis Products LLC	Mylan NV	N/A	Undisclosed		Completed
2000	2000-05-25	Actavis Pharma Inc	Allergan plc		891.44	Stock	14.08	Completed
2000	2000-08-04	PDK Labs Inc	PDK Acquisition Corp		19.04	Cash	3.39	Completed
1999	1999-08-10	Copley Pharmaceutical Inc	Teva Pharmaceutical Industries Ltd		214.68	Cash	14.12	Completed
1998	1997-06-03	Faulding Inc	Mayne Pharma International Pty Ltd		103.25	Cash		Completed
1997	1996-12-25	Royce Laboratories Inc	Allergan plc		N/A	Stock		Completed

1996	1996-01-29	Biocraft Laboratories Inc	Teva Pharmaceutical Industries Ltd		356.1	Stock		Completed
1995	no deals							

YEAR	ANNOUNCED VALUE (MIL)	ANNOUNCED VALUE (BIL)	# OF DEALS
2016	42946.82	42.95	17
2015	26661.10	26.66	16
2014	885.00	0.89	7
2013	67.50	0.07	6
2012	2235.65	2.24	4
2011	1106.48	1.11	7
2010	1252.87	1.25	5
2009	9.63	0.01	6
2008	10481.85	10.48	6
2007	1864.27	1.86	7
2006	7623.89	7.62	5
2005	4193.75	4.19	11
2004	648.98	0.65	9
2003	0.00	0.00	5
2002	0.00	0.00	2
2001	150.00	0.15	3
2000	910.48	0.91	2
1999	214.68	0.21	1
1998	103.25	0.10	1
1997	0.00	0.00	1
1996	356.10	0.36	1
1995	0.00	0.00	0
**Total**	**101712.30**	**101.71**	**122**
